# Defining the window of opportunity and target populations to prevent peanut allergy

**DOI:** 10.1016/j.jaci.2022.09.042

**Published:** 2023-05

**Authors:** Graham Roberts, Henry T. Bahnson, George Du Toit, Colin O’Rourke, Michelle L. Sever, Erica Brittain, Marshall Plaut, Gideon Lack

**Affiliations:** a University of Southampton and Southampton NIHR Biomedical Research Centre, Southampton, and the David Hide Centre, Isle of Wight, United Kingdom; b Benaroya Research Institute and the Immune Tolerance Network, Seattle, Wash; c Pediatric Allergy Group, Department of Women and Children’s Health, School of Life Course Sciences, King’s College, and the Children’s Allergy Service, Guy’s and St Thomas’ NHS Foundation Trust, London, United Kingdom; d Rho Federal Systems Division, Durham, and PPD Government and Public Health Services, Wilmington, NC; e National Institute of Allergy and Infectious Diseases, Bethesda, Md

**Keywords:** Peanut allergy, prevention, diet, early introduction, population

## Abstract

**Background:**

Peanut allergy affects 1% to 2% of European children. Early introduction of peanut into the diet reduces allergy in high-risk infants.

**Objective:**

We aimed to determine the optimal target populations and timing of introduction of peanut products to prevent peanut allergy in the general population.

**Methods:**

Data from the Enquiring About Tolerance (EAT; n = 1303; normal risk; 3-year follow-up; ISRCTN14254740) and Learning Early About Peanut Allergy study (LEAP; n = 640; high risk; 5-year follow-up; NCT00329784) randomized controlled trials plus the Peanut Allergy Sensitization (PAS; n = 194; low and very high risk; 5-year follow-up) observational study were used to model the intervention in a general population. Peanut allergy was defined by blinded peanut challenge or diagnostic skin prick test result.

**Results:**

Targeting only the highest-risk infants with severe eczema reduced the population disease burden by only 4.6%. Greatest reductions in peanut allergy were seen when the intervention was targeted only to the larger but lower-risk groups. A 77% reduction in peanut allergy was estimated when peanut was introduced to the diet of all infants, at 4 months with eczema, and at 6 months without eczema. The estimated reduction in peanut allergy diminished with every month of delayed introduction. If introduction was delayed to 12 months, peanut allergy was only reduced by 33%.

**Conclusions:**

The preventive benefit of early introduction of peanut products into the diet decreases as age at introduction increases. In countries where peanut allergy is a public health concern, health care professionals should help parents introduce peanut products into their infants’ diet at 4 to 6 months of life.

## Graphical abstract

**Figure:**
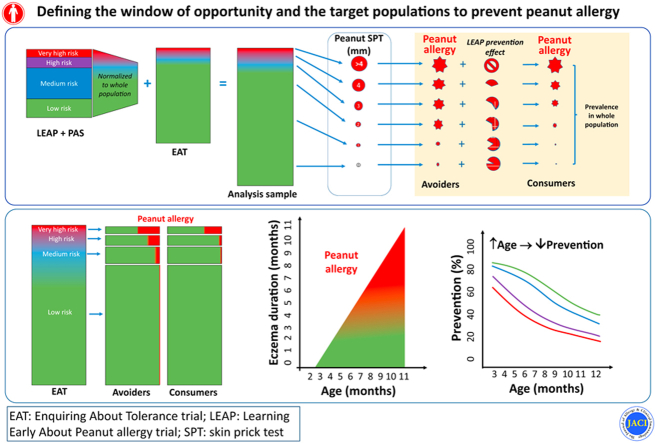

Peanut allergy represents an important health burden affecting 1% to 2% of North American and European children,^1^^,^^2^ with considerable impact on quality of life.^3^, ^4^, ^5^, ^6^ The Learning Early About Peanut Allergy (LEAP) trial demonstrated that early introduction of peanut in a high-risk population of infants can reduce their risk of peanut allergy at age 5 years by 81%.^7^^,^^8^ However, we note that 76 of 834 infants in the LEAP screening study could not be enrolled because they had a skin prick test (SPT) wheal result of >4 mm and therefore had likely already developed peanut allergy.^9^

The 2017 US National Institute of Allergy and Infectious Diseases–sponsored prevention guideline advocated introducing peanut into the infant diet at 4 to 6 months for those with severe eczema or egg allergy, around 6 months for those with mild-to-moderate eczema, and at an age-appropriate time in accordance with family preferences and cultural practices for other infants.^10^ However, these recommendations were based on expert opinion, extrapolating from a high-risk population.^11^ More recently, the 2021 European Academy of Allergy and Clinical Immunology prevention guideline suggests introducing peanut into the infant diet at 4 to 6 months in populations where there is a high prevalence of peanut allergy.^12^ The guideline also highlighted that understanding the effectiveness of the early introduction of peanut products across the whole population is a high-priority gap in our evidence base. Moreover, it should be noted that since the change in Australian guidelines in 2016, consumption of peanut during the first year of life increased from 28.4% before the guidelines (2007-11) to 88.6% after the implementation of the guidelines (2016-18).^13^ Despite this change, a recent publication has shown no decline in the observed prevalence of peanut allergy in Australia in 2020, which remained stable at 3.1%.^14^

Here we detail an analysis that aimed to assess the impact of the early introduction of peanut into the infant diet on the prevention of peanut allergy across the whole population and may partially explain why the rate of peanut allergy in Australia has not decreased. First, we assessed which readily identifiable factors were associated with developing peanut allergy in the first year of life. Different risk profiles may limit the effectiveness of the intervention by narrowing the window of opportunity in which peanut allergy can be prevented.^8^ Second, we modeled the relative reduction in peanut allergy that is likely to occur at 5 years of life depending on when peanut is introduced into the diet in the whole population.^15^ We assume that the prevalence of peanut allergy in the Enquiring About Tolerance (EAT) trial at age 3 years is a predictive surrogate of peanut allergy at 5 years. This modeled approach provides an assessment of the intervention’s effectiveness across a whole population and across different risk strata according to the month of life that peanut is introduced into an infant’s diet.

## Methods

### Study design

This study utilized published data from the LEAP screening study,^9^ published and unpublished data from the LEAP randomized controlled prevention trial,^7^ unpublished data from the Peanut Allergy Sensitization (PAS) observation study, and published data from the EAT randomized controlled prevention trial (see Fig E1 in this article’s Online Repository at www.jacionline.org).^16^ Together, the 4 studies covered a broad swath of risk factors for peanut allergy observed across a normal population. EAT provides information about low-risk individuals, while the LEAP screening study, the LEAP randomized controlled trial (RCT), and the PAS observation study provide information about high-risk and very high-risk individuals. The analysis makes use of individual participant-level data, and combining the data sets allows many cases of peanut allergy to be modeled across different cohorts and risk levels. The approach taken made several clearly identified assumptions, which are described and justified in Table E1 in the Online Repository.

### Participants and interventions

All data for the analyses presented are available this article’s Methods section in the Online Repository at www.jacionline.org.

#### LEAP screening study

The LEAP screening study was the recruitment phase of the LEAP trial.^7^ Full details have been published elsewhere.^9^ Briefly, recruitment targeted infants between 4 and 11 months of age with severe eczema, egg allergy, or both. Participants were separated into 4 groups: group I (low-risk PAS study) had mild or no eczema and no egg allergy (exclusion criteria for LEAP); group II (LEAP-negative stratum) had severe eczema and/or egg allergy but no reaction on SPT to peanut; group III (LEAP-positive stratum) had severe eczema and/or egg allergy and a 1 to 4 mm peanut wheal; and group IV (high-risk PAS study) had severe eczema and/or egg allergy and peanut wheal responses of >4 mm (exclusion criteria for LEAP), which we refer to here as “likely allergy” (see Table E2 in the Online Repository at www.jacionline.org).

#### LEAP prevention trial

The LEAP trial randomized 640 infants, aged 4 to 11 months with severe eczema, egg allergy, or both, to early peanut introduction or avoidance during early life. These participants encompassed the LEAP screening study groups II and III; each of these 2 cohorts was independently powered, randomized, and analyzed.^7^ The LEAP trial determined that peanut allergy was prevented in the early introduction group within both cohorts (Table E2).^7^^,^^17^

#### PAS study

The PAS study comprised 2 subgroups of participants who were not eligible for inclusion onto the LEAP trial (Table E2).^9^ LEAP screening group I was considered too low risk to be enrolled, and LEAP screening group IV was considered likely already allergic on the basis of SPT wheal sizes of >4 mm. These participants did not receive the LEAP intervention; however, they were followed up at 60 months of age and assessed for clinical allergy using the same LEAP trial protocol.^7^

#### EAT trial

The published EAT trial evaluated whether the early introduction of 6 allergenic foods into the diet of breast-fed infants would protect against the development of food allergy.^16^ Briefly, the EAT trial recruited, from the whole population of the United Kingdom, 1303 exclusively breast-fed infants (age 3 months) (Table E2). Participants were randomized to the early introduction of 6 allergenic foods (peanut, cooked egg, cow’s milk, sesame, whitefish, and wheat; early introduction group) or to exclusive breast-feeding to 6 months of age (standard introduction group). The primary outcome was food allergy to 1 or more of the 6 foods at 1 to 3 years of age.

### Data analysis

#### Assessing factors associated with development of peanut allergy during the first year of life

In order to stratify the risk of peanut allergy during the first year of life and target populations for early prevention strategies, we selected key risk factors predictive of peanut allergy that could be readily screened for during a public health intervention. These key risk factors were ethnicity, eczema severity, eczema duration, and age. Baseline peanut allergy was defined by oral food challenge (LEAP and EAT, early introduction groups) or peanut SPT wheal >4 mm at the baseline or 1-year visit (other groups) (see Table E1 and the Methods section in the Online Repository at www.jacionline.org).^18^, ^19^, ^20^, ^21^

#### Estimating the impact of early introduction of peanut to the whole population and to different risk groups

To assess the impact of the early introduction of peanut into the infant diet in a normal-risk population with good adherence to the intervention, the prevalence of peanut allergy at 36 months in the early introduction group was estimated by applying the relative reduction of peanut allergy observed with the LEAP intervention in <15, 15-40, and >40 Severity Scoring of Atopic Dermatitis (SCORAD) bands in the LEAP trial.

#### Estimating the impact of early peanut introduction at different ages to the whole population

To model the whole population using combined EAT, LEAP, and PAS study data, data of LEAP and PAS participants were weighted such that the overall distribution of eczema severity, egg allergy, and non-White ethnicity would match the normal EAT population using propensity scores (see, in this article’s Online Repository available at www.jacionline.org, the Methods section and Fig E2). These weights were applied in an ordinal logistic regression model of SPT wheal size category at each month of age with peanut avoidance (see Fig E3 in the Online Repository).

A logistic regression model was used to estimate the prevalence of allergy at 5 years of life depending on peanut SPT wheal size and subject age in the first year of life with peanut avoidance (Fig E3). The LEAP intention-to-treat (ITT) intervention effect was estimated by logistic regression (see the Methods section in the Online Repository at www.jacionline.org), where this effect represents the reduction in allergy if introducing peanut conditional on each SPT wheal size during the first year of life versus avoiding peanut until age 5 (see Fig E4 in the Online Repository).

The LEAP intervention effect was applied, stratified by age and peanut SPT wheal size, to determine the prevalence of allergy at 5 years of age, under both strategies using different approaches (see the Methods section in the Online Repository available at www.jacionline.org) to estimate the relative reduction of peanut allergy by age at intervention.

Analyses were performed by R v4.0.2 (R Project; www.r-project.org), JMP Pro 15 (SAS Institute, Cary, NC), and SAS v9.4 software.

## Results

The EAT, LEAP, and PAS study participants are described in Fig E5 in the Online Repository at www.jacionline.org. Together, they covered the entire range of eczema severity (see Fig E6 in the Online Repository).

### Early introduction to peanut

The impact of early introduction is not as effective among all participants screened in LEAP because many already had peanut allergy. Early introduction of peanut in the LEAP study resulted in an 81% reduction in peanut allergy at 60 months of age in the ITT analysis (Table I).^7^ Many participants were excluded from LEAP because they had likely had peanut allergy by 4 to 11 months of age, when the intervention was applied.^18^^,^^19^ If all participants in the LEAP screening study had received the intervention, then the overall reduction would have been 52% (Table I).

**Table I:** Impact of early peanut introduction on allergy in the LEAP screening cohort

LEAP screening study group	Sample size	Peanut allergy in avoidance group at 60 months of age	Peanut allergy in early introduction group at 60 months of age	Reduction in each group	Reduction in LEAP trial participants
I (low risk)	118	0.8%∗	NA	NA†	
II (high risk)	542	13.7%	1.9%	86.1%	81.0%
III (high risk sensitized)	98	35.3%	10.6%	70.0%	
IV (likely peanut allergic)	76	81.4%	NA	NA‡	
All groups	834	20.4%			

The LEAP screening cohort includes 2 groups (groups II and III), and 2 other groups, a high-risk and a low-risk group, which were not included in the RCT. Group IV (n = 76) was considered already allergic (peanut SPT wheal of >4 mm). Group I (n = 118) had mild eczema and no egg allergy, and was considered too low risk to be entered into the trial. Groups II and III were randomized to early introduction or avoidance of peanut. All groups were assessed for peanut allergy by the same method at 60 months. *NA,* Not applicable.

∗ Participants in group I not assessed at 60 months were assumed to be not peanut allergic.

† Intervention not applied.

‡ Intervention not applicable because subjects were assumed to already be allergic. If groups I and IV had received the intervention (and if we assume complete benefit in group I and no benefit in group IV), then the reduction in peanut allergy across the LEAP screening cohort (groups I-IV) would be 52% ([(0.019 × 542) + (0.106 × 98) + (1 × 76)]/[118 + 542 + 98 + 76]/ [(0.137 × 542) + (0.353 × 98) + 1.000 × 76]/[118 + 542 + 98 + 76)]), rather than the 81% seen in the LEAP trial.

### Factors associated with peanut allergy during infancy

#### Increasing age or longer duration and eczema severity are related to likelihood of peanut allergy in the first year of life

**Fig 1:**
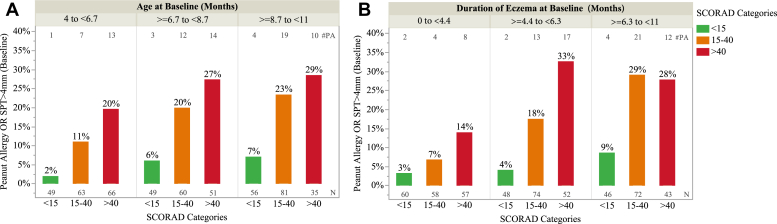
Relationship between age at baseline and reported duration and eczema severity on the likelihood of peanut allergy at baseline in the first year of life. *Bars* represent prevalence of peanut allergy at baseline (raw data), defined by baseline oral food challenge or SPT wheal of >4 mm at screening, for participants in the LEAP screening cohort (7 LEAP RCT and 76 PAS group IV participants). Participants aged 4 to 11 months were assessed in the study at baseline and defined as low risk (all group I subjects, assumed to be tolerant), high risk and high risk sensitized (groups II and III from early introduction group, assessed by baseline peanut challenge), and likely allergy (group IV, assumed to be peanut allergic, with a peanut wheal of >4 mm) (Table E1). Those randomized to peanut avoidance (groups II and III) were omitted because they were not assessed for peanut allergy by oral food challenge at baseline. Proportion of infant peanut allergy by **(A)** tertile of age at screening (months) and **(B)** tertile of duration of eczema at screening (months); duration was the more important risk factor (Fig E7). The number with baseline peanut allergy is annotated *above* each bar, and the sample size is *below* each bar.

In the LEAP screening study, the likelihood of peanut allergy at the time of baseline assessment increased with increasing age and eczema severity (Fig 1, *A*). There was a similar relationship between peanut allergy and increasing duration of eczema (Fig 1, *B*), with duration being the more important risk factor (see Fig E7 in the Online Repository at www.jacionline.org).

#### Diameter of SPT wheal increases with age during infancy, and most who develop peanut allergy by 5 years have allergy by 12 months

**Fig 2:**
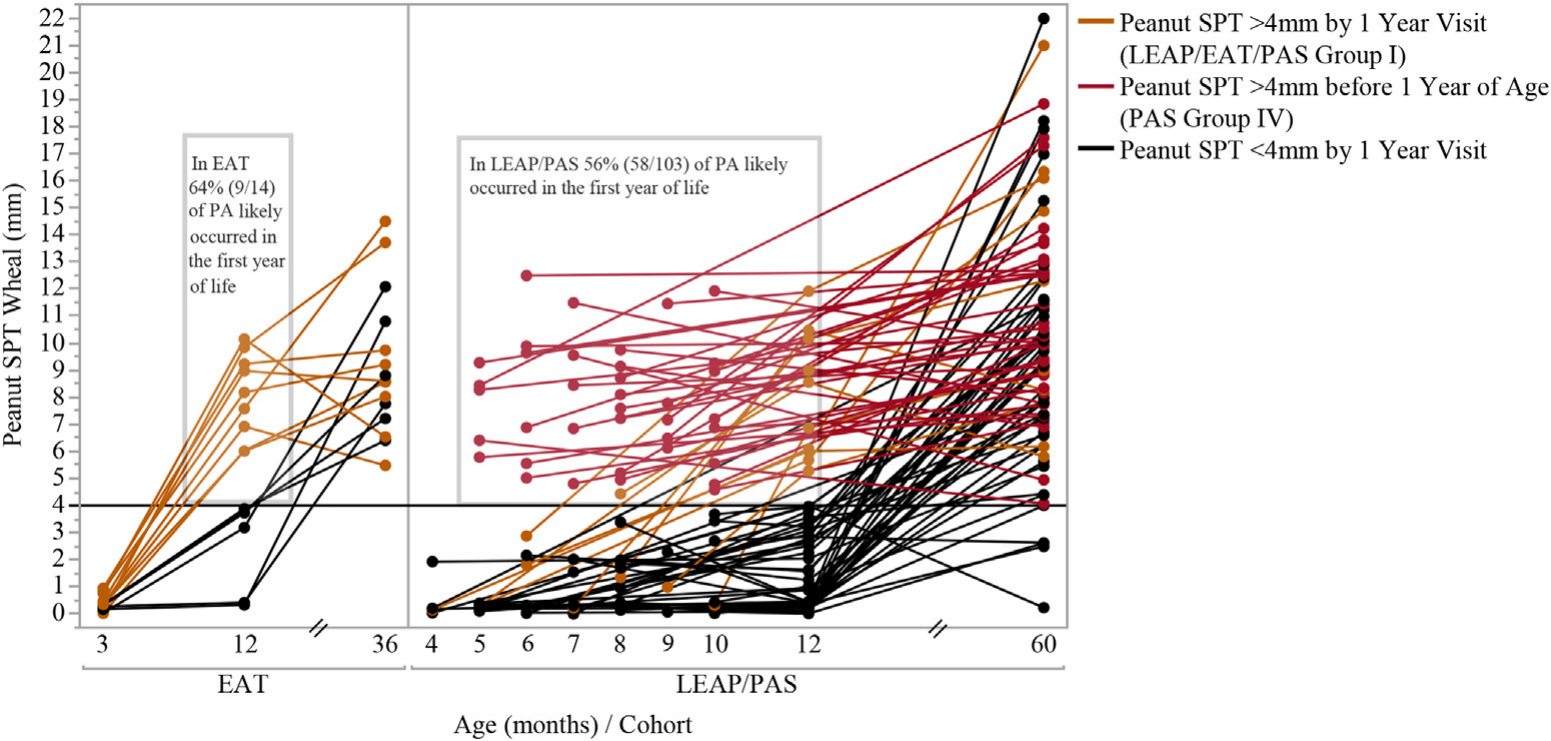
Trajectory of peanut wheal sizes of avoidance group participants allergic to peanut at the final assessment (n = 53; 36 months for EAT and 60 months for LEAP and PAS participants). Each *line* represents an allergic participant’s SPT values over the course of the study, starting with their age (in months) at baseline. SPT was not collected in the EAT avoidance group at 3 months; therefore, a distribution was imputed on the basis of the EAT early introduction group SPT distribution at baseline. Because 99% of SPT distribution at 3 months in the EAT early introduction group was between 0 and 1 mm, points were jittered within this interval so that lines could be connected between the 3-, 12-, and 36-month assessments. Participants with a >4 mm wheal at screening are identified with *red lines* (PAS group IV) and only had SPT data available at the screening visit and the 60-month visit. *Orange lines* represent EAT and LEAP allergic, avoidance group participants whose wheal sizes were >4 mm by their 12-month visit. *Black lines* represent allergic participants from the avoidance group whose wheal sizes were <4 mm by their 12-month visit. Assuming that participants with an SPT wheal of >4 mm are allergic to peanut,^18^, ^19^, ^20^, ^21^ approximately 60% of participants with peanut allergy at the end of the study were allergic at or before their 12-month visit based on wheal sizes of >4 mm. *PA,* Peanut allergy.

Data from the high-risk LEAP screening and normal-risk EAT studies showed that participants who were older at screening were more likely to present with a bigger SPT wheal to peanut (see Fig E8 in the Online Repository at www.jacionline.org), with none sensitized at <5 months of age. Looking longitudinally at avoidance participants, the SPT wheal diameter of those who ultimately developed peanut allergy increased rapidly during the first year of life (Fig 2), with the most allergic manifesting at 12 months (peanut SPT wheal >4 mm, highly predictive of allergy^18^, ^19^, ^20^, ^21^) (Table E1).

#### Non-White ethnicity is associated with greater development of peanut allergy during the first year of life

Combining EAT and LEAP cohort data revealed that non-White (including mixed ethnicity) infants were estimated to have a higher likelihood of peanut allergy compared to White infants (relative risk 2.22, 95% confidence interval 1.45-3.33, *P* < .001) (see the Methods section and Fig E9 in the Online Repository at www.jacionline.org).

### Estimating the impact of early introduction of peanut to the whole population and to different risk groups

#### Potential impact of applying the LEAP intervention to EAT, a normal-risk population

The adherence to early introduction of peanut in the infant diet was poor in the normal population EAT study. If adherence was similar to that seen in the LEAP study, then peanut allergy prevalence would have reduced from 2.5% to 0.29%. If the LEAP intervention was targeted exclusively at infants with severe eczema (SCORAD > 40) at greatest risk, the total population burden of peanut allergy would be reduced by <5%. Targeting the larger number of children with mild eczema (30% reduction) or no eczema (29% reduction) has much greater impact (Table II).

**Table II:** Prevalence and population burden of peanut allergy at 36 months by SCORAD bands and the potential impact of applying the LEAP intervention to EAT, a normal-risk population

Eczema risk groups by SCORAD	Proportion of EAT avoidance group (n)	Peanut allergy at 36 months		Peanut allergy burden (proportion of total allergy in avoidance group by stratum)
		Avoidance group (observed data from EAT)	Early introduction group	
>40	0.5% (3)	33.3%	10.32%	6.64%
15-40	4.9% (29)	13.8%	0.69%	25.58%
1-14	18.5% (110)	4.6%	0.55%	33.61%
0	76.2% (454)	1.1%	0.13%	33.17%
All	100% (596)	2.5%	0.29%	

Observed proportions of peanut allergy in the EAT avoidance group are shown for each eczema risk stratum.^16^ Prevalence of peanut allergy at 36 months in the early introduction group was estimated by applying the relative reduction of peanut allergy observed with the LEAP intervention for that SCORAD band (see Fig E11 in the Online Repository at www.jacionline.org). The burden of peanut allergy explained by each stratum takes into account the size of the risk stratum and the allergy rate within each stratum. If the intervention was applied only to the >40 (severe eczema), 15-40 (moderate eczema), 1-14 (mild eczema), or 0 SCORAD bands, then the population burden of peanut allergy would be reduced by 4.55%, 25.43%, 29.65%, or 29.20%, respectively.

#### Estimating the impact of early introduction of peanut at different ages to the whole population

The estimation of treatment effect by timing in the whole population depends on a number of assumptions, so a few simpler estimates were also assessed to ensure the robustness of our whole-population model.

**Fig 3:**
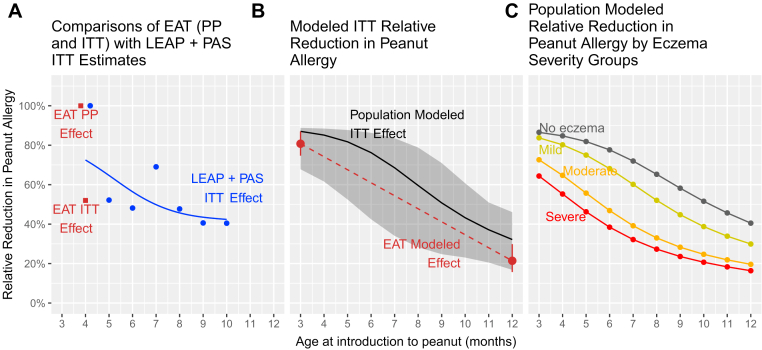
Relative reduction in burden of peanut allergy in a normalized population by age at introduction for **(A)** raw data from each study, **(B)** EAT-modeled effect plus whole-population model, and **(C)** whole-population model by eczema severity. All relative reductions estimate the treatment effect between early peanut introduction and avoidance. *(A)* EAT ITT and per-protocol (PP; restricted to only those exposed to the intervention) point estimates are displayed as *red squares* and are calculated as relative reductions between the standard introduction and early introduction arms. The *blue points* and *blue smoothed regression line* using a spline term for age shows relative reduction estimates from the raw high-risk LEAP screening population data (that is, LEAP + PAS, with imputed treatment effect among the PAS cohort, where the imputed benefit in PAS group IV was 0). *(B) Red dashed line* shows EAT-modeled estimates using the LEAP ITT treatment effect (Fig E4) applied at 3 months and 12 months. The whole-population (EAT + LEAP + PAS)-modeled ITT effect with bootstrapped 95% confidence intervals is shown in *black and gray* (see Fig E14 in the Online Repository at www.jacionline.org for sensitivity analyses). *(C)* The whole-population–modeled ITT effect is shown by eczema severity. Additional sensitivity analyses and modeling details relevant to these analyses are shown in the Online Repository (see Figs E12 and E13, and Tables E4, E5, and E6).

We first estimated the effect of early introduction by age at first introduction for the observed results from EAT (ITT and per-protocol effect) and the combined LEAP + PAS data set (ITT effect) where no or minimal assumptions are required (Fig 3, *A*). The impact decreased with increasing age at introduction. Second, the impact on the normal-risk EAT population at 3 and 12 months was modeled using the LEAP effect size (Table E1), showing similar results (Fig 3, *B*).

Then we replicated the estimation of the impact of introducing peanut into the infant diet at different ages using our whole-population model (Fig 3, *B*). Full details, including our assumptions, are included in Table E1 and the Methods section in the Online Repository available at www.jacionline.org. The bootstrapped confidence intervals indicate a decreasing relative reduction of peanut allergy with increasing age at introduction to peanut. The negative impact of delaying the introduction of peanut into the diet was most apparent in infants with increasing eczema severity (Fig 3, *C,* and see Fig E12, *B,* in the Online Repository) and/or non-White ethnicity (Fig E12, *C* and *D*).

We calculated the combined effect of intervening at different ages in infants with and without eczema on the peanut allergy burden in the total population. We chose 3 different illustrative scenarios: (1) introduction of peanut to infants with and without eczema at 4 months resulted in an 82% relative reduction in peanut allergy; (2) introduction in infants with eczema at 4 months and without eczema at 6 months resulted in a 77% risk reduction; and (3) introduction in infants with eczema at 4 months and at 12 months in infants with no history of eczema resulted in a 58% relative risk reduction (see Table E3 in the Online Repository at www.jacionline.org) relative to peanut avoidance.

## Discussion

The LEAP trial findings have resulted in a fundamental shift in our approach to peanut allergy prevention.^22^ They have now been replicated in both the UK EAT and Scandinavian PreventADALL RCTs.^16^^,^^23^ We sought to evaluate the impact of timing the introduction of peanut products into different risk groups during infancy in a general population to reduce the burden of peanut allergy. In both the LEAP screening cohort and EAT trial, we found that the majority of peanut allergy had already developed by the first year of life (Fig 2), especially among those with severe eczema, egg allergy, and non-White ethnicity (Fig 1, Fig 2, Fig 3, and see Fig E15 in the Online Repository at www.jacionline.org). Confining the intervention to the highest risk infants has a minimal impact on the overall population burden; the greatest benefit was achieved when the whole population is targeted, as the majority of peanut allergy occurs in the large lower risk groups (Table II). The impact of the early introduction of peanut products was most effective when applied as early as possible. This reflects the experience in Israel, a culture in which peanut products are commonly introduced early into the infant diet and peanut allergy is rare.^24^

Our analysis demonstrating the need to intervene at the whole-population level agrees with previous publications extrapolating data from the LEAP trial. O’Connor et al^25^ estimated that if the intervention was applied only to Irish infants with severe eczema and egg allergy, the population burden of peanut allergy would only have been reduced by 29%. Similarly, Koplin et al^18^ in an Australian cohort estimated that targeting the intervention to infants with severe eczema and/or egg allergy would have reduced the population disease burden by only 6%, which is similar to our estimate (Table II). Applying simple, low-cost, safe interventions to the whole population is a more effective preventive public health strategy than targeting selected groups.^26^ Last, there is a theoretical consequence that introducing peanut exclusively to high-risk infants may result in a greater environmental peanut exposure of lower risk infants who are not consuming peanut. This could result in a higher rate of peanut allergy in this lower risk group that is not protected by early peanut consumption, as predicted by the dual allergen exposure hypothesis.^27^

Over several decades, the deliberate avoidance of peanut has understandably led to parental fear of early introduction. Applying early introduction of peanut to a whole population requires considerable education of health care professionals and families, with detailed advice on weaning strategies and addressing families’ concerns. The safety of early introduction of peanut products has been observed in LEAP and EAT.^16^^,^^28^ We need to be aware of unintended consequences,^29^ such as the possibility of parents’ giving infants whole nuts, leading to a risk of nut inhalation. It is critical that education stresses the need to introduce peanut products such as a peanut butter or peanut puffs—not the whole nut.

We have shown that in both a high-risk and normal population, the majority of peanut allergy has already developed in the first year of life (Fig 2). This aligns with the Australian HealthNuts cohort, where 3.1% of infants had challenge-proven peanut allergy at 1 year of age.^2^^,^^30^ The 3.1% number is similar to the overall peanut allergy rate expected in the Australian population. A recent US publication also confirms that a high rate of challenge proven peanut allergy is seen in the first year of life, at 18% in infants with moderate to severe eczema, which is similar to that seen in LEAP.^31^ Additionally, infants under 6 months of age had a much lower likelihood of having peanut allergy compared to those over 6 months, even with severe eczema. In their series of 321 infants aged 4 to 11 months whose parents responded to publicity about the study, twice as many as in the LEAP screening study would have defined as already having peanut allergy by the LEAP study criteria.^9^ This highlights the necessity for early intervention. Although our results may not be exactly applicable to all populations, it is reassuring from the PreventADALL study that early introduction of peanut products was able to significantly prevent peanut allergy in an RCT trial in Sweden and Norway.^23^ The easily identifiable factors in early infancy that are associated with early development of peanut allergy are severity and duration of eczema plus non-White ethnicity, which could be used to identify high-risk infants (Fig 1, Fig E8, and Fig E12, *C* and *D*). The important question regarding whether age at introduction of peanut into the diet affects the strategy’s efficacy has previously been raised.^32^ Our analysis of only the RCT cohorts of the LEAP study found that the intervention was equally effective in younger and older infants.^33^ However, when the entire LEAP screening study cohort is assessed, older age at introduction reduces the efficacy (Fig 3, *A*). This is because some of the infants developed peanut allergy early in infancy, before the intervention could have commenced, and thus were excluded from the LEAP RCT (Figs 2 and 3). Also, the intervention itself was less effective in children with increasing wheal diameters to peanut (Fig E4), and we observed that wheal size increased with age (Figs 2 and 3; and see Fig E10 in the Online Repository at www.jacionline.org).

Our modeled approach, consistent with the raw data, points to the need for early intervention by 6 months of age for the whole population, with even earlier intervention from 4 months of age in those with eczema (Fig 3, *C*). This reflects the relatively narrow window of opportunity to prevent peanut allergy, which appears to be most time-critical in infants with eczema (especially severe eczema) and in UK non-White infants (Fig E9). A simpler approach would be to recommend early introduction of peanut products to all children by 6 months of age, but this would fail to prevent the development of allergy in a substantial proportion of infants with eczema (Fig E12, *B*).

This analysis provides meaningful insight into the benefits of early introduction of peanut because it uses RCT data including participants with all levels of risk of developing peanut allergy as well as follow-up data from participants who did not fulfill the LEAP entry criteria. Additionally, this analysis has challenge-proven primary outcomes for most participants, and all of the studies had high completion rates (89%). However, our analysis has some limitations. In generating the population model, several assumptions are made, which are highlighted and justified in Table E1. One important assumption is the LEAP treatment effect for each risk group was used in our modeled approach. However, this treatment effect may be a conservative estimate, given the very high per-protocol effect sizes in both the LEAP and EAT trials (98% and 100% relative reduction, respectively).^7^^,^^16^ The LEAP and EAT trials differed in how the intervention was applied and in the length of follow-up, so the preventative effect may have been underestimated in EAT as a result of the potential for some resolution of allergy from 3 to 5 years of age. In some analyses, we have used an SPT wheal of >4 mm as indicative of allergy, given that there are published data suggesting that 75% of these infants have peanut allergy.^18^, ^19^, ^20^, ^21^ These data used the same SPT solutions (ALK-Abelló, Hørsholm, Denmark) and methodology as the LEAP and EAT cohorts, and our diagnostic assumptions are presented in detail in Table E1 and Figs E1 and E3. Another potential criticism is that the EAT participants were all exclusively breast-fed until at least 3 months of age—a narrower population than the full UK general risk group. A systematic review has concluded that breast-feeding is not associated with food allergy^34^; additional analysis in the LEAP study did not show a significant effect of breast-feeding on the intervention’s efficacy (Table E1).

As acknowledged, our whole-population model (Fig 3, *B*) relies on assumptions, and furthermore, there are inherent vulnerabilities associated with linking the multiple data sources. Therefore, it is reassuring that the much more simply estimated treatment effect by age in the combined LEAP/PAS high-risk analysis (Fig 3, *A*) has a similar slope to the modeled general population curve (Fig 3, *B*), as did the modeled treatment effect in the EAT study (Fig 3, *B*). That said, the LEAP/PAS sensitivity analyses include the possibility of a substantial decrease in benefit between 4 and 5 months, followed by a relatively smaller decline between 5 and 8 months (see point estimates in Fig 3, *A,* and see Fig E13 in the Online Repository at www.jacionline.org).

We have generated a model for the burden of peanut allergy across a whole UK population. Our estimates show that it is most advantageous to intervene in the whole population. If we were to introduce peanut products in high-risk infants with any eczema at 4 months of age and in all other infants at 6 months of age, we estimate that we could reduce the burden of peanut allergy in the population by 77%. This provides evidence for the recommendations in the recent North American and European guidelines that suggest the early introduction of peanut products for all infants on the basis of an extrapolation from previously published evidence from the LEAP and EAT studies.^12^^,^^35^ We would advocate that public health policies should recommend that peanut products are introduced at 4 to 6 months of age in countries where peanut is an important allergen. Health care professionals supporting families with introducing complementary feeding should encourage introduction at 4 months when eczema is present. Support will be needed to help families know when their infant is ready for solids and to help them choose the most appropriate peanut product. Encouragingly, data now indicate that 88.6% of Australia infants are consuming peanut in the first year of life following changes to the country’s national infant feeding guidelines (2016).^13^ While this prevention strategy appears to have practically influenced behavior in a real-world setting, disappointingly, the rate of peanut allergy has remained stable at 3.1%.^14^^,^^36^ Interestingly, we report that earlier introduction, especially less than 6 months of age compared to after 12 months of age, is significantly associated with a substantially reduced risk of peanut allergy among those of Australian ancestry. Our findings both support and explain these observations while emphasizing the need for earlier introduction to prevent peanut allergy in the general population.

**Clinical implication**

To maximize the prevention of peanut allergy in the population, all infants should start eating peanut products by 6 months of life; infants with eczema, especially severe eczema, should start from 4 months of age.
