# Supplementary material for: Defining the window of opportunity and target populations to prevent peanut allergy
Source: J Allergy Clin Immunol. 2023 May;151(5):1329–36. doi: 10.1016/j.jaci.2022.09.042 (PMC10689252; doi:10.1016/j.jaci.2022.09.042)
Supplement: Supplementary data [file mmc1.pdf]

Online supplement

Defining the window of opportunity and the target populations to prevent peanut allergy

Authorship: Graham Roberts*, D.M., Henry T. Bahnson*, M.P.H., George Du Toit, M.B., B.Ch., Colin O'Rourke, M.S., Michelle L. Sever, Ph.D., Erica Brittain, PhD, Marshall Plaut, M.D., Gideon Lack, FRCPCH.

*Equal contribution

The authors' affiliations:

Graham Roberts: University of Southampton and Southampton NIHR Biomedical Research Centre, Southampton, and the David Hide Centre, Isle of Wight, UK

Henry T. Bahnson: Benaroya Research Institute and the Immune Tolerance Network, Seattle, USA

George Du Toit: Pediatric Allergy Group, Department of Women and Children's Health, School of Life Course Sciences, King's College London; the Children's Allergy Service, Guy's and St Thomas' NHS Foundation Trust, London, UK

Colin O'Rourke: Benaroya Research Institute and the Immune Tolerance Network, Seattle, USA

Michelle L. Sever: Rho Federal Systems Division, Chapel Hill, USA; PPD Government and Public Health Services, Wilmington, USA

Erica Brittain: The National Institute of Allergy and Infectious Diseases, Bethesda, USA

Marshall Plaut: The National Institute of Allergy and Infectious Diseases, Bethesda, USA

Gideon Lack: Pediatric Allergy Group, Department of Women and Children's Health, School of Life Course Sciences, King's College London; the Children's Allergy Service, Guy's and St Thomas' NHS Foundation Trust, London, UK

Corresponding author:

Gideon Lack, MB, BCh, FRCPCH, Children's Allergy Service, 2nd Floor, Stairwell B, South Wing, Guy's and St Thomas' NHS Foundation Trust, Westminster Bridge Rd, London SE1 7EH, United Kingdom.

E-mail: gideon.lack@kcl.ac.uk.

CONTENTS

1. METHODS

A. Design 3

B. LEAP screening study 3

C. LEAP prevention study 7

D. Peanut Allergy Sensitization Study (PAS) 8

E. EAT study 10

F. Assessing factors associated with peanut allergy during infancy 10

G. Estimating the impact of early introduction of peanut 11

H. Role of the funding source 17

2. RESULTS

A. Participants from EAT and LEAP and PAS studies 18

B. Factors associated with peanut allergy during the first year of life 18

C. Estimating the impact of early introduction of peanuts to the whole population 24

3. FIGURES

E1. Modelling approach using data from EAT, LEAP and PAS cohort to estimate the effect of timing of the intervention on its impact in the whole population 4

E2. Distribution of propensity scores between EAT and LEAP 12

E3. Probability of peanut allergy at 36/60 months conditional on SPT and age in the first year of life 13

E4. Modeled ITT intervention effect in LEAP by SPT size at baseline 14

E5. Consort figure showing LEAP screening, LEAP and EAT studies 19

E6. Distribution of participants with no, mild, moderate and severe eczema in each study population during infancy 20

E7. Combined effect of eczema severity and duration and age on the likelihood of peanut allergy at the screening visit in LEAP 20

E8. Relationship between duration of eczema and age with the likelihood of peanut allergy or sensitization during the first year of life 21

E9. Time to event modeling of the development of allergy in the first year of life based on SPT > 4mm 22

E10. Distribution of peanut wheal diameters by age in the first year of life 23

E11. Relative reduction in peanut allergy with LEAP intervention by SCORAD group 25

E12. Population modeled ITT relative reductions in peanut allergy and bootstrapped 95% confidence intervals for (A) all participants and (B) by eczema severity (C and D) ethnicity 26

E13. Estimated relative reductions in peanut allergy and bootstrapped 95% confidence intervals in the LEAP and PAS cohorts 28

E14. Sensitivity analyses of different approaches used to weight LEAP+PAS to a normal population (EAT) 29

E15. Association between infant peanut allergy and eczema severity and egg allergy at baseline (LEAP/PAS) and 1 year (EAT) 30

4. TABLES

E1: Assumptions used to extrapolate to whole population 5

E2: Study populations included in this analysis 9

E3: Simulations of relative reductions in peanut allergy based on timing of intervention scenarios 31

E4. EAT observed allergy rates (ITT and PP) 31

E5: LEAP + PAS modeled allergy rates (ITT) 32

E6: EAT modeled allergy rates (ITT) 32

5. REFERENCES 33

1. METHODS

A. Design

The design of the study is summarized in **Figure E1**. Datasets from the LEAP and EAT trials were combined with the goal of reflecting the whole UK population. The LEAP and PAS data sets were standardized to match the risk factor representation in the reference general population, EAT. Standardization used the key predictors of infant peanut allergy: eczema severity, ethnicity and egg allergy. This modeled population was used to estimate the impact of the early introduction of peanut into the infant diet across different risk strata and age groups. Assumptions are listed in **Table E1**.

B. LEAP screening study

The LEAP screening study was the recruitment phase of the LEAP trial.[1] Full details have already been published[2] with the key details reproduced below.

Study design

The LEAP screening study was a single-center, prospective, observational study conducted at a single site in the United Kingdom that included infants who underwent screening for the LEAP interventional trial,[1,3] which investigated the prevention of peanut allergy in high-risk children.

Participants

Recruitment was targeted to families with young infants with severe eczema, egg allergy, or both. Recruitment occurred from December 2006 to May 2009. It focused on (i) child health professionals, such as dermatologists, allergists, and specialist nurses; (ii) a study flyer posted to parents of young infants in the United Kingdom; and (iii) other avenues, such as written and electronic media and word of mouth. Interested families were asked to make contact with the study. Infants underwent screening for the LEAP study if they passed a prescreening questionnaire addressing previous allergy and eczema history. Screening included an assessment of eczema severity, egg allergy and skin prick test (SPT) sensitization to peanut.

Severe eczema was defined as one of the following: (i) frequent need for treatment with topical corticosteroids or calcineurin inhibitors, (ii) parental description of ‘‘a very bad rash in joints and creases’’ or ‘‘a very bad itchy, dry, oozing, or crusted rash,’’ or (iii) a severe SCORAD grade (>40)[4] by a clinician before or at the time of screening. Participants were split into 3 eczema severity groups based on SCORAD scores (mild, <15; moderate, 15-40; and severe, >40).

Egg allergy was defined on the basis of either (i) an SPT-induced wheal diameter of 6 mm or greater with raw hen’s egg white and no history of previous egg tolerance or (ii) an SPT-induced wheal diameter of 3 mm or greater with pasteurized hen’s egg white with a history of an allergic reaction to egg.

Allergic sensitization assessment

SPTs to ingested allergens, including raw hen’s egg white (Red Lion salmonella-free egg), pasteurized hen’s egg white, peanut, cow’s milk, sesame, and soya (all other SPTs sourced from ALK-Abello, Hørsholm, Denmark), were undertaken at the baseline assessment. Using a standardized lancet (ALK-Abello), the skin on the forearm was pricked through a drop of the extract. Peanut SPTs were undertaken in duplicate, with the widest diameter of the wheals at 15 minutes recorded and averaged. A saline control was not subtracted.

Participant groups

We categorized all infants who were screened into groups of increasing atopy (**Table E2**):

- Group I, with ‘‘mild eczema and no egg allergy,’’ did not meet LEAP trial inclusion criteria.
- Group II, with ‘‘severe eczema and/or egg allergy but no reaction on SPT to peanut,’’ is the LEAP trial negative SPT response stratum.
- Group III, with ‘‘severe eczema and/or egg allergy and a 1-4 mm peanut wheal,’’ is the LEAP trial positive SPT response stratum.

Figure E1. Modelling approach using data from EAT, LEAP and PAS studies to estimate the effect of timing of the intervention on its impact in the whole population

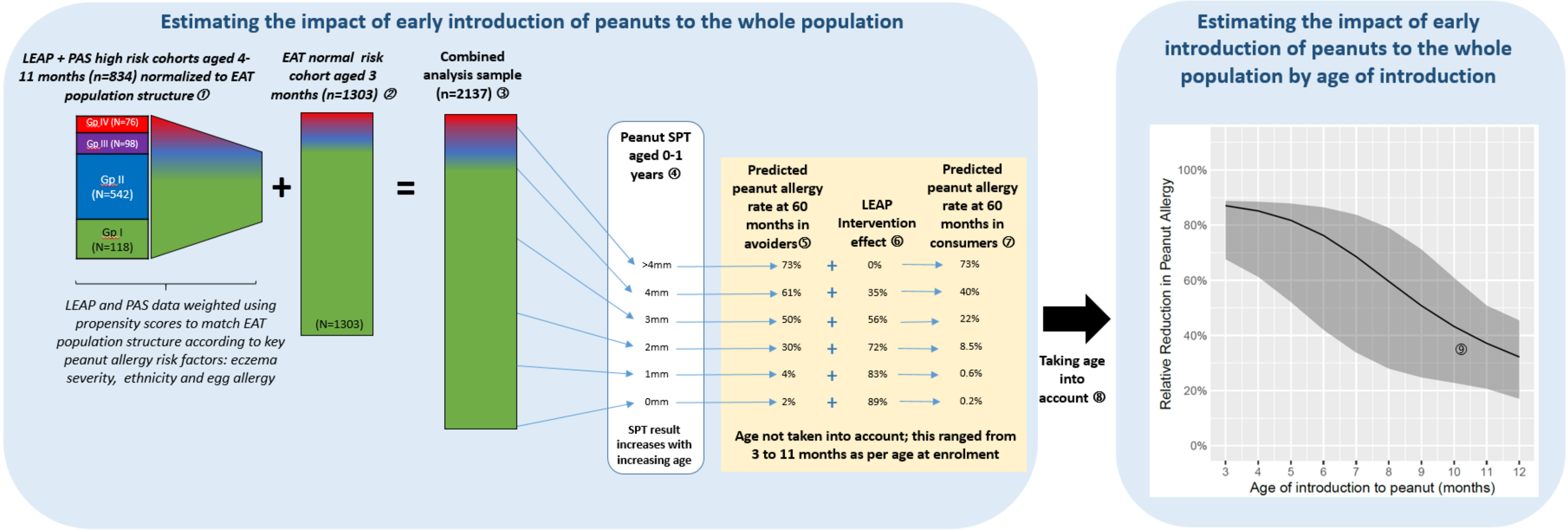

Concept figure illustrating the approach taken in this study to model the effect of early introduction of peanut into the infant diet across the whole of the population. *Estimating the impact of early* introduction *of peanuts in the whole population:* LEAP and PAS high risk cohorts (①) normalized to reflect the general population in terms of eczema severity (SCORAD bands 0, 1-14, 15-39, ≥40), ethnicity and egg allergy prevalence as described by EAT (②). A logistic regression model using eczema severity, ethnicity and egg allergy was used to estimate propensity scores to weight the combined analysis sample and represent a general population(③). These propensity score weights were then used in an ordinal logistic regression model to estimate the proportion of peanut SPT wheal sizes of 0,1, 2, 3, 4 or >4mm during infancy in a general population (④). The prevalence of peanut allergy in the avoiders was computed by a logistic model conditional on SPT and age (⑤) and consumers (⑦) by assuming the LEAP intervention effect computed using a logistic regression model with an interaction effect between peanut SPT size and randomized treatment assignment (⑥) (**Figure E4**). *Estimating the impact of the early* introduction *of peanuts to the whole population by age of introduction:* This took into account the age of infants when peanut would be introduced into the diet (⑧). The impact of the intervention when applied at different ages was assessed taking into account the different proportion of infants with 0, 1, 2, 3, 4 or >4mm peanut SPT wheals at each month of age (⑨). See online methods section G for more details.

Table E1. Assumptions used to extrapolate to whole population

Main assumptions	Justifications and data limitations
1) The EAT trial is representative of the UK general population in terms of risk of peanut allergy	We need to consider eczema prevalence and severity as these are the principal risk factors for peanut allergy. The prevalence of eczema at 3 months in the EAT avoidance arm was 24.1%. There is a lack of published literature on the prevalence of eczema in the first few years of life. The BASELINE cohort was recruited at a similar time to EAT. At 6 months of age, 18.7% (299 of 1597) of the BASELINE infants were diagnosed with eczema on the basis of the UK Working Party diagnostic criteria.[5]<br>The EAT trial participants were all exclusively breast fed at enrolment.[6] A 2014 systematic review concluded that there is no clear or consistent evidence that breastfeeding affects the development of food allergy.[7] The efficacy of the intervention is also equivalent in breast and non-breast fed infants as there is an 83% reduction in peanut allergy in breast fed infants and an 80% reduction in non-breast fed LEAP infants. Also, this effect size, in a population where breastfeeding was mixed and variable, is very similar to the breastfeeding effects in per protocol EAT infants who were exclusively breast fed for at least 3 months.[6]
2) Peanut allergy in 3 year old children (EAT time point) is a reasonable surrogate for their allergy status at age 5 years (LEAP/PAS time point).	We have assessed peanut allergy at 3 years in EAT and at 5 years in LEAP/PAS. Our data indicate that the prevalence of peanut allergy is fairly stable between 3 and 5 years of age. In the LEAP study, 42/309 (13.6%) of participants had a SPT >4mm at 30 months compared to 54/316 (17.1%) at 60 months of age. Furthermore, the Australian HealthNuts study showed that most allergy had already developed by 1 year of age.[8,9]<br>In LEAP strict avoidance of peanut in the avoidance arm was recommended until 5 years of age while in the EAT study avoidance was only recommended during the first year. In practice, both avoidance groups avoided peanut throughout the trial follow up.[1,6] Therefore, differences in study design in EAT versus LEAP/PAS (age of final assessment and control group regimen) were not impactful.
3) Peanut SPT>4mm indicates peanut allergy in infants	A total of 357 oral peanut challenges were undertaken in the HealthNuts study in infants aged 11-15 months.[8,9] Peanut challenges were positive in 148. The investigators present data indicating that a peanut wheal of >4mm equates to 75% probability of challenge proven peanut allergy (Peters et al, 2013, Figure 2).[8] Using data from a more recent article[9] we further tested the robustness of our assumptions that a peanut SPT >4mm indicates peanut allergy in infants. Specifically, we calculated that a diagnostic cut-off >4mm afforded 93% sensitivity, 91% specificity, and 91% accuracy in predicting challenge proven peanut allergy at 12 months of life in their cohort. We also developed a logistic regression model using the continuous wheal size measurements in their data and then applied this model to the 1-year SPT distribution in our dataset (as shown in our online supplement, **Figure E10**). The output from this model was a predicted probability of allergy for each 'likely allergic' subject based on the logistic regression model trained on the Koplin et al data.[9] The median predicted probability of allergy was 84%, which is very similar to the empirical estimates we derived from our own data as shown in supplemental **Figures E1 and E3**. Moreover, in a smaller previous Australian case series with 18 participants aged

<table>
<tr><td></td><td>0-2 years, all those with a >4mm wheal (n=14) had peanut allergy on the basis of a positive challenge, indicating a 100% PPV.[10] Finally, Santos et al have published data on children (43 with challenge proven peanut allergy, 36 who were sensitized to peanut, and 25 with no sensitization) where the positive predictive value of a >4mm wheal was 77%.[11] So we have made a reasonable assumption that all participants with >4mm peanut wheal already have clinical allergy and so will not be susceptible to this prevention strategy.</td></tr>
<tr><td>4) Additional assumptions for fully modelled estimates (illustrated in Figures 4B and 4C):
a) Considering the LEAP's intervention effect model applied to LEAP, PAS and EAT avoidance rates:
i) The treatment effect models the intervention as a function of SPT, and only SPT
ii) The treatment effect model, which conditions only on SPT, applies to children at lower risk
iii) No benefit for peanut SPT>4mm
iv) The peanut allergy rates in the EAT standard introduction arm reasonably estimates the rates for avoidance.
b) Considering standardization of LEAP/PAS to match EAT:
i) In addition to ethnicity, the cohorts were matched on the basis of baseline eczema severity and egg allergy. The measurement of these variables at different baseline ages in the different cohorts has such a small effect that it can be ignored</td><td>i) Peanut SPT wheal diameter is the best surrogate for clinical allergy. Although the final model estimating the treatment effect size uses only SPT, a second model estimates the SPT distribution using age, eczema severity, ethnicity, and egg allergy. Although the raw data suggest there is a sharper decrease in efficacy for SPT>2mm then the model estimates, a sensitivity analysis indicates little impact on the conclusions due to this difference.
ii) The lower risk children are individuals with no or mild eczema. An analysis of the raw data from the LEAP study demonstrates that there was a 88% reduction in peanut allergy in participants with no or mild eczema (Figure E11).
iii) Participants that had SPT>4mm (Group IV) were likely to already have peanut allergy at enrolment.[8-11] It was therefore assumed to be too late to prevent the development of peanut allergy.
iv) The control rates in the EAT group may be slightly lower because some peanut introduction occurred before 5 years; however this is unlikely to have much effect on the relative reduction estimates.
i) Key risk factors for peanut allergy are eczema, egg allergy, and ethnicity. We therefore postulate that by matching for these variables, we can standardize the higher risk LEAP/PAS population to the UK general risk EAT population. Because the study designs were inherently different with respect to age at baseline, we need to assume, for example, that a child who enrolls with egg allergy at 3 months has similar risk for peanut allergy as a child who enrolls with egg allergy at 8 months. Despite this challenge, the weighted model using the combined cohorts gives a 2.3% overall prevalence of peanut allergy which is as expected from the literature.[12] This prevalence calculation was done as follows: Using the ordinal model of wheal size, first estimating the distribution across SPT categories at an average age in the weighted sample under an avoidance strategy [P(SPT = 0) = 97.9%, P(SPT = 1) = 0.531%, P(SPT = 2) = 0.323%, P(SPT = 3) = 0.34%, P(SPT = 4) = 0.181%, P(SPT > 4) = 0.681%]. Second we estimate the prevalence of allergy in the avoidance group at an average age at each SPT size [P(Allergy | SPT = 0) = 1.44%, P(Allergy | SPT = 1) = 2.66%, P(Allergy | SPT = 2) = 27.6%, P(Allergy | SPT = 3) = 49.7%, P(Allergy | SPT = 4) = 62%, P(Allergy | SPT > 4) = 75.5%]. We multiply these to get the joint distribution of allergy and wheal size. Ultimately, we find that the [P(Allergy & SPT = 0) = 1.41%, P(Allergy &</td></tr>
</table>

ii) Some individuals in the LEAP/PAS cohort were assigned very high weights to achieve a matched distribution to the EAT cohort; it is assumed that this did not materially bias estimates	SPT = 1) = 0.0141%, P(Allergy & SPT = 2) = 0.0893%, P(Allergy & SPT = 3) = 0.169%, P(Allergy & SPT = 4) = 0.113%, P(Allergy & SPT > 4) = 0.514%], which, when summed across SPT, gives a marginal allergy prevalence of 2.31%.<br>ii) As described above our weighting approach gave estimates that are concordant with the literature. In addition, several sensitivity analyses shown in **Figure E14** examined the impact of high weights, which reinforced the robustness of the chosen weighting schema used in terms of implications to when to introduce peanut.

C. LEAP prevention trial

The LEAP trial aimed to evaluate whether peanut introduction during early life prevented the development of peanut allergy in infants at high risk for peanut allergy. Participants have been followed up at 60[1] and 72[3] months. Only data to 60 months is utilized in this study. Full details have already been published[1] with the key details reproduced below.

Study design

The LEAP trial was a randomized, open-label, controlled trial conducted at a single site in the United Kingdom designed to investigate whether early introduction of peanuts into the infant diet would prevent the development of peanut allergy in high-risk children.

Participants

Participants were enrolled from the LEAP screening study (described above). Participants in Groups II and III were eligible (**Table E2**). This gave two strata divided on the basis of peanut SPT: Group II ‘‘severe eczema and/or egg allergy but no reaction on SPT to peanut (LEAP study negative SPT response stratum) and Group III with ‘‘severe eczema and/or egg allergy and a 1-4 mm peanut wheal (LEAP study positive SPT response stratum). Each of these two strata were independently powered to allow the intervention to be assessed separately in peanut sensitized and non-sensitized infants.

Intervention

Participants in each LEAP trial stratum were then randomly assigned to a group in which dietary peanut would be consumed or a group in which peanut would be avoided. Infants randomly assigned to early introduction underwent a baseline, open-label food challenge in which those who had had negative SPT results were given 2 g of peanut protein in a single dose and those who had positive SPT results were given incremental doses up to a total of 3.9 g. Participants who had a reaction to the baseline challenge were instructed to avoid peanuts but were included in the intention-to-treat analysis. Participants randomly assigned to early introduction who did not have a reaction to the baseline challenge were fed at least 6 g of peanut protein per week, distributed in three or more meals per week, until they reached 60 months of age. The preferred peanut source was Bamba, a snack food manufactured from peanut butter and puffed maize. Smooth peanut butter (the brands Sunpat or Duerr’s) was provided to infants who did not like Bamba. Participants assigned to avoidance were to avoid the introduction of peanut protein until they reached 60 months of age. Adherence was assessed with the use of a validated food frequency questionnaire.

Trial outcomes

Clinical assessments were undertaken at baseline (when participants were between 4 and 11 months of age) and at the ages of 12, 30, and 60 months.

The primary outcome was the proportion of participants with peanut allergy at 60 months of age and was determined in 617 participants by means of an oral food challenge. Participants in whom peanut allergy was unlikely (no wheal after SPT at months 30 and 60, no history of allergic symptoms after ingestion of peanut, no diagnosis or suspicion of allergies to sesame or tree nut, and no history of anaphylaxis in response to any food) received 5 g of peanut protein in a single dose. A double-blind, placebo-controlled food challenge was conducted for other participants (with a total of 9.4 g of peanut protein administered in increments) in accordance with standard dose-escalation procedures. Among 11 study participants for whom data from the oral food challenge were either inconclusive or not available, a diagnostic algorithm based on clinical history, the results of a SPT, and the values for peanut-specific IgE were used to determine whether or not a participant should be considered to have peanut allergy.

At baseline and at all study visits, SPT for peanut allergy were performed in duplicate with the use of a lyophilized peanut extract (manufactured by ALK-Abello), and the average of the diameter of the two widest wheals was recorded.

Statistical analysis

Primary statistical analyses were performed in each stratum independently on an intention-to-treat basis with data from all participants who could be assessed for the primary outcome. The analyses compared the proportion of participants with peanut allergy in the peanut-avoidance group with the proportion with peanut allergy in the early peanut introduction group at month 60 with the use of a two-tailed chi-square test. A per protocol analysis was also performed including participants who adhered adequately to the assigned regimen until 2 years of age. Analyses of the data from the two cohorts were independently powered. Datasets for the LEAP trial are available through TrialShare, a public website managed by the Immune Tolerance Network (www.itntrialshare.org/LEAP.url). Analyses were performed using R version 4.0.2 (Vienna, Austria), JMP Pro 15, and SAS 9.4 (Cary, NC).

Ethical considerations

Ethical approval for the study was provided by the NRES Committee London – Fulham, formerly West London REC2 Ethics Committee (REC Reference 04/Q0403/13). Informed consent was obtained from the parents of all participants.

D. Peanut Allergy Sensitization (PAS) study

Study design

The PAS study comprised the participants who were not eligible for randomization in the LEAP trial (**Table E2**).

Participants

The PAS study comprised of two LEAP screening study groups who were followed-up at 60 months. Group I participants were not sufficiently atopic to be included in the LEAP trial as they had no egg and milk allergy or no severe eczema. Of the 118 eligible participants 47 were followed-up at 60 months of age. Group IV participants had skin prick test results to peanut >4mm between 4-11 months of life and were assumed to already have peanut allergy. Of the 76 eligible participants 59 were followed-up at 60 months of age.[2]

Study outcomes

The PAS study made an assessment of all consenting participants that could be reached in Group I and group IV at 60 months of age; the first visit was on 11 November 2011 and the last visit was on 01 July 2015. At the single study visit, participants underwent the same evaluation that participants in the LEAP study undergo at 60 months of age.

Statistical analysis

The clinical and immunological data obtained from the PAS participants at 5 years of age was entered into the LEAP database. The study database was locked in February 2018. The analyses focused on the proportion of participants with peanut allergy. The primary outcome datasets for the PAS study are available through TrialShare, a public website managed by the Immune Tolerance Network (www.itntrialshare.org/LEAP.url).

Ethical considerations

Ethical approval for the study was provided by the London Westminster Research Ethics Committee (11/LO/0045). Informed consent was obtained from the parents of all participants.

Table E2. Study populations included in this analysis

Study	EAT trial (n=1303)	LEAP screening study			
		**Group I (PAS study) Low risk (n=118)**	**Group II (LEAP trial) High risk (n=542)**	**Group III (LEAP trial) High risk-sensitized (n=98)**	**Group IV (PAS study) Likely allergy (n=76)**
Design	Normal population, randomized controlled prevention cohort	Low risk observational cohort	Moderate risk randomized controlled prevention cohort	High risk randomized controlled prevention cohort	Very high risk observational cohort
Age at inclusion	3-4 months	4-11 months	4-11 months	4-11 months	4-11 months
Inclusion criteria	Plan to breast feed until 6 months of age.	Mild to moderate eczema, egg allergy, or both. Successfully weaned onto at least one solid.	Severe eczema, egg allergy, or both. Peanut SPT 0mm Successfully weaned onto at least one solid.	Severe eczema, egg allergy, or both. Peanut SPT 1-4mm Successfully weaned onto at least one solid.	Eczema (usually severe), egg allergy, or both. Peanut SPT >4mm. Successfully weaned onto at least one solid.
Exclusion criteria	Chronic illness, prematurity	Chronic illness, previous or current introduction of peanut, suspected peanut allergy, household member with peanut allergy.			
Intervention	Peanut, egg, milk, sesame, fish, soy from 3 months of age or avoidance	None	Introduction of peanuts into diet at 4-11 months or avoidance		Recommended peanut avoidance given SPT result to peanut of >4mm
Age of assessments of SCORAD and SPT	3, 12, 36 months	4-11, 60 months	4-11, 12, 30, 60 months		4-11, 60 months
Peanut challenge	36 months	60 months	60 months	60 months	60 months

EAT study is a general population study.[7] The LEAP study participants[1] represents the LEAP screening study[2] participants who were eligible for the peanut allergy prevention trial. Groups I and IV represent infants who were too mild or too severe, respectively, for entry into the LEAP prevention study;[2] they were followed up in the PAS study. Sensitization defined on the basis of a 3mm or greater wheal on skin prick testing. Peanut allergy defined on the basis of double-blind, placebo-controlled challenges. Peanut consumption defined as occasional (<3g peanut protein per week), infrequent (3-5g peanut protein per week) or frequent (≥6g peanut protein per week).

E. EAT trial

The EAT trial evaluated whether the early introduction of allergenic foods in the diet of breast-fed infants would protect against the development of food allergy. Full details have already been published[6,12] with key details reproduced below.

Study design

The EAT trial was a randomized, controlled trial, conducted at a single site in the United Kingdom.

Participants

Enrollment took place from November 2009 to July 2012. Singleton infants who were 3 months of age and exclusively breast-fed were recruited from the general population in England and Wales (**Table E2**). Breastfeeding is not known to influence the prevalence of either eczema or peanut allergy.

Intervention

Participants were randomly assigned by an independent online service to the standard -introduction group or the early introduction group. Participants in the standard introduction group were to be exclusively breast-fed to approximately 6 months of age. After 6 months of age, the introduction of allergenic foods was allowed according to parental discretion. Participants in the early introduction group had six allergenic foods introduced: cow's milk (yogurt) first, followed (in random order) by peanut, cooked (boiled) hen's egg, sesame, and whitefish; wheat was introduced last. Infants in the early-introduction group who had a wheal of any size on skin-prick testing at baseline (standard introduction group did not have SPT at baseline) underwent an open-label incremental food challenge totaling 2 g of protein of that food. Families of infants in the early introduction group who had negative results on skin-prick testing or who had positive results on skin-prick testing but negative results on the food challenge were asked to continue feeding their infants 2 g of the allergen protein twice weekly. Families of infants who had a positive result on the food challenge at baseline were instructed to avoid giving the infants that food but to continue feeding the infants the other foods.

Trial outcomes

All the families completed an online questionnaire each month to 1 year of age, and then every 3 months until the child reached 3 years of age. This questionnaire recorded the frequency of consumption of allergenic foods in the two groups. Participants had scheduled assessments at 1 year of age and 3 years of age.

The primary outcome was challenge-proven food allergy to one or more of the six early introduction foods between 1 year and 3 years of age.

Statistical analysis

The intention-to-treat analysis for the primary outcome included all the participants who had data that could be evaluated. The analysis, which compared the proportion of participants in the two groups who had food allergy to one or more of the early introduction foods, was performed with a chi-square test. A per protocol analysis was also undertaken that included all participants who adhered adequately to the assigned regimen. The data set for the EAT trial is available through TrialShare, a public website managed by the Immune Tolerance Network (www.itntrialshare.org).

Ethical considerations

Ethical approval was provided by the St. Thomas' Hospital research ethics committee (reference 08/H0802/93). Written informed consent was obtained from parents or guardians.

F. Assessing factors associated with peanut allergy during infancy

Logistic regression analysis

To define the best target population for prevention, it is important to know which individuals in the whole population are likely to develop peanut allergy in infancy. Therefore, the risk factors for infant peanut allergy were assessed using data from LEAP trial[2] (moderate and high risk groups II and III early introduction arms with their baseline peanut challenges), PAS groups I (low risk with negative peanut SPT so assumed to be tolerant at screening) and IV (with

peanut SPT >4mm diameter so assumed to already be allergic at screening based on previous data (**Table E1**)[8-11]) and/or EAT (intervention only as control group did not have baseline SPT).

Baseline peanut allergy was defined by oral food challenge at the baseline visit within the LEAP early introduction group (4-11 months of age) and the EAT early introduction group (3 months of age). For the remaining groups, a peanut skin prick test wheal >4mm at the baseline or 1 year visit was used to define 'likely allergy' (**Table E1**).[8-11] Skin prick testing in EAT was not undertaken in the standard introduction (avoidance) group at the baseline visit because the results could have influenced the timing of introduction of allergenic foods.

Baseline eczema severity, eczema duration, age and ethnicity (white or non-white) were included. Egg allergy was not included as its status is often unknown in early infancy and therefore has less utility for public health interventions. The association between age, eczema severity, and peanut allergy in infancy was explored using a logistic regression model. EAT data were not included for duration of eczema analysis as the range of ages and durations of eczema at baseline were limited to 3 months based on the enrollment criteria.

Sensitivity analysis - time to event model

In addition to modeling with logistic regression, a population weighted, Weibull time to event model was developed to look at the risk of developing infant peanut allergy during the first year of life and its relation to eczema severity and ethnicity. This sensitivity analysis uses SPT wheal size greater than 4 mm as a surrogate measure for allergy, since allergy status is only recorded at the end of the study (3 and 5 years of age). All patients were assumed to be non-allergic up until 3 months of age. Participants who were not yet allergic at 12 months of age or who entered into the early introduction group were considered right censored, the former at 12 months and the latter at the time they began the intervention. For those who became allergic, due to the sparse nature of follow-up, the exact time at which a participant became allergic is unavailable. These were then considered interval-censored event times, where the censoring interval lies between the baseline enrollment age and the 12-month allergy assessment.

G. Estimating the impact of early introduction of peanuts into the whole population and different risk groups.

Estimating the impact of early introduction at different ages to the whole population

Modelling the whole population using combined EAT, LEAP and PAS study data

The three cohorts were combined. In order to model the whole population, LEAP and PAS participants were weighted such that the overall distribution of eczema severity, egg allergy, and non-white ethnicity would match the distribution in the general EAT population. This general population was estimated from weighted logistic regression models using direct standardization with propensity scores (**Figure E2**).[13]

EAT was assumed to be a general population in terms of eczema severity (SCORAD bands 0, 1-14, 15-39, ≥40), ethnicity and egg allergy prevalence (see ②in **Figure E1**). Using EAT as the reference population, a propensity score weighted ordinal logistic regression model was developed, so that the weighted LEAP+PAS cohort would have equal distributions on the specified risk factors to the EAT cohort (see ①). Key risk factors and inclusion criteria were used to develop the propensity scores (i.e. using eczema severity groups, ethnicity (white or non-white) and egg allergy), where assigned weights are 1 for EAT and $p_i/(1-p_i)$ for LEAP+PAS, where p_i is the propensity score for the ith subject (**Figure E2**). Due to the inherent differences in study designs, assessments of eczema and egg allergy were made at different ages for EAT than for LEAP+PAS, and it is assumed that these differential assessment times had little impact on the estimation of the weights.

Figure E2. Distribution of propensity scores between EAT and LEAP studies

(A)

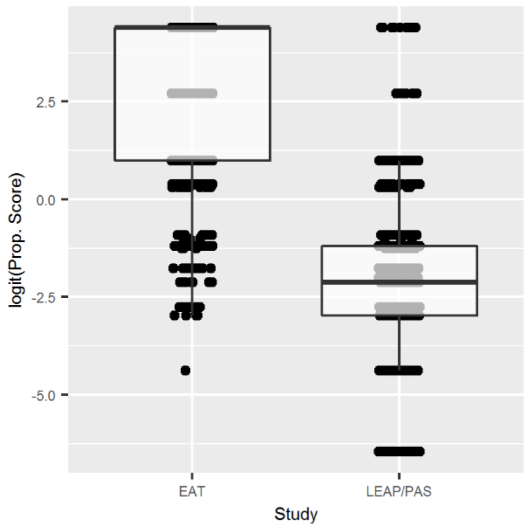

(B)

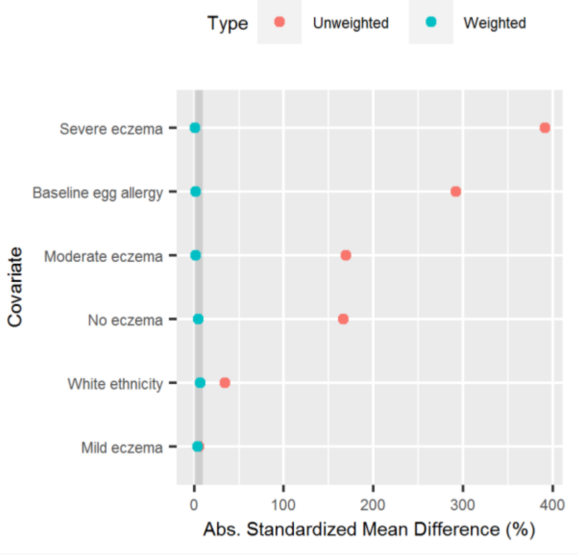

(C)

	Level	Unweighted		Weighted	
		EAT	LEAP/PAS	EAT	LEAP/PAS
Baseline age (months)		3.00 (0.00)	7.26 (1.78)	3.00 (0.00)	7.28 (2.05)
Baseline egg allergy	Yes	0.04 (0.18)	0.57 (0.49)	0.04 (0.18)	0.04 (0.19)
Ethnicity	White	0.85 (0.36)	0.72 (0.45)	0.85 (0.36)	0.82 (0.38)
Baseline Eczema severity	None	0.76 (0.43)	0.04 (0.20)	0.76 (0.43)	0.74 (0.44)
	Mild	0.19 (0.39)	0.21 (0.40)	0.19 (0.39)	0.20 (0.40)
	Moderate	0.05 (0.22)	0.42 (0.49)	0.05 (0.22)	0.05 (0.23)
	Severe	0.01 (0.08)	0.33 (0.47)	0.01 (0.08)	0.01 (0.09)

Propensity scores were computed to create weights for each observation in the LEAP/PAS cohorts such that both cohorts would be balanced with respect to baseline egg allergy, ethnicity, and eczema severity. (A) Shows estimated propensity scores relative to the EAT trial based on age, ethnicity, egg allergy, and eczema severity. The distributions are clearly different owing to the different risk profiles of each population and there are some extreme weights associated with some participants. Nevertheless, there is overlap among the cohorts, and this provides evidence that the data sources can be weighted and combined for analysis. To consider the impact of the extreme weights and different "trimmed weighting strategies" sensitivity analyses were performed. These are shown in **Figure E14** and reinforce the primary strategy chosen for the analyses presented in **Figure 3**, and the advantage of early introduction is robustly illustrated across these sensitivity analyses. (B) Shows the degree of balance obtained by weighting the LEAP/PAS participants by the propensity score weights. This shows that by weighting much of the imbalance of these covariates between the studies is removed. The x-axis displays the standardized mean difference, which is the difference in means between the EAT sample and the LEAP sample, divided by the standard deviation in the EAT group. For example, the prevalence of egg allergy is 3.5% in EAT and 56.8% in LEAP. Taking this difference and dividing by the standard deviation is: (0.035 – 0.568)/0.183 = -2.91. This is expressed as a percentage of a standard deviation. Therefore, the difference between the EAT and LEAP samples with respect to egg allergy is 291% before weighting. However, after weighting this figure is about 2% of a standard deviation. (C) This table summarizes covariate balance before and after weighting. Means (or proportions) and standard deviations before and after applying weighting are shown. We acknowledge that the difference in baseline age illustrates an inherent weakness of this weighting that is unavoidable due to different study designs. Thus, even though there is an excellent balance on baseline risk factors, we need to assume, for example, that a child who enrolled at 8 months with egg allergy has approximately the same risk for peanut allergy as a child who enrolled at 3 months with egg allergy.

Figure E3. (A) Probability of peanut allergy at 36/60 months and (B) weighted (left number) and unweighted (right number) proportions of the sample conditional on SPT and age in the first year of life

(A)

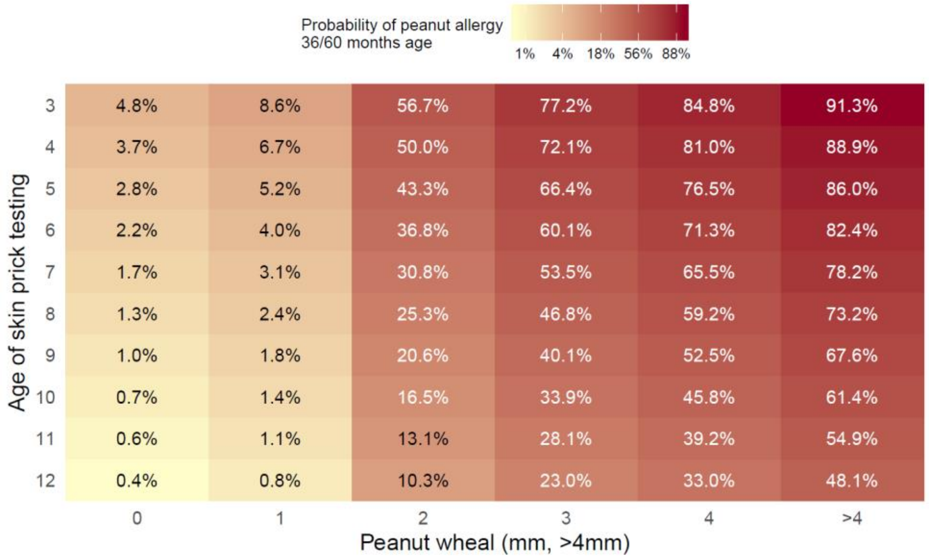

(B)

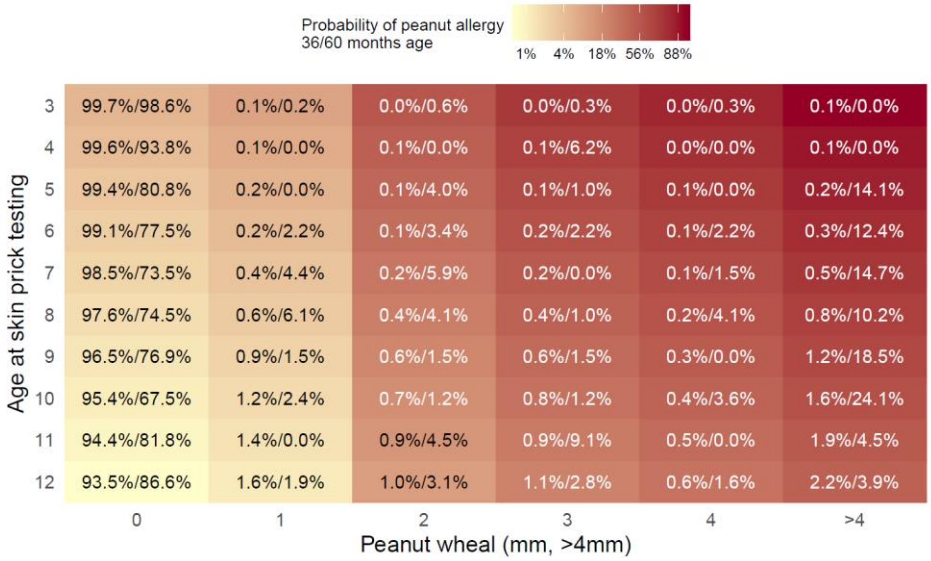

This figure presents component pieces of the whole population model for a cohort without peanut consumption: the probability of allergy at 5 years as a function of SPT and age it was measured and the distribution (shown in A), and the distribution of SPT by age (shown in B). (A) The color corresponds to the probability of allergy from the weighted logistic regression model of the combined cohorts described in section 1G above. (B) Shows the distribution of the weighted and unweighted sample conditional on age and SPT size. Specifically, the left proportion gives the row percentage of subjects within each age using the propensity score weighted proportional odds logistic regression model and the right proportion gives the proportions out of the unweighted analysis sample. The probabilities in (A) were combined with the estimated prevalence of each peanut wheal size occurring in the first year of life in (B) to give the overall probability of peanut allergy at each age over the first year of life. The LEAP intervention effect, shown in **Figure S4** below, was then used to model the relative reduction in peanut allergy, shown in **Figure 3** of the main manuscript. In combination, **Figures E3A, E3B, and E4** give the raw estimates used to calculate the relative risks shown in **Figure 3**.

Figure E4. (A) Modeled and (B) observed ITT intervention effect in LEAP by SPT size at baseline

(A) **(B)**

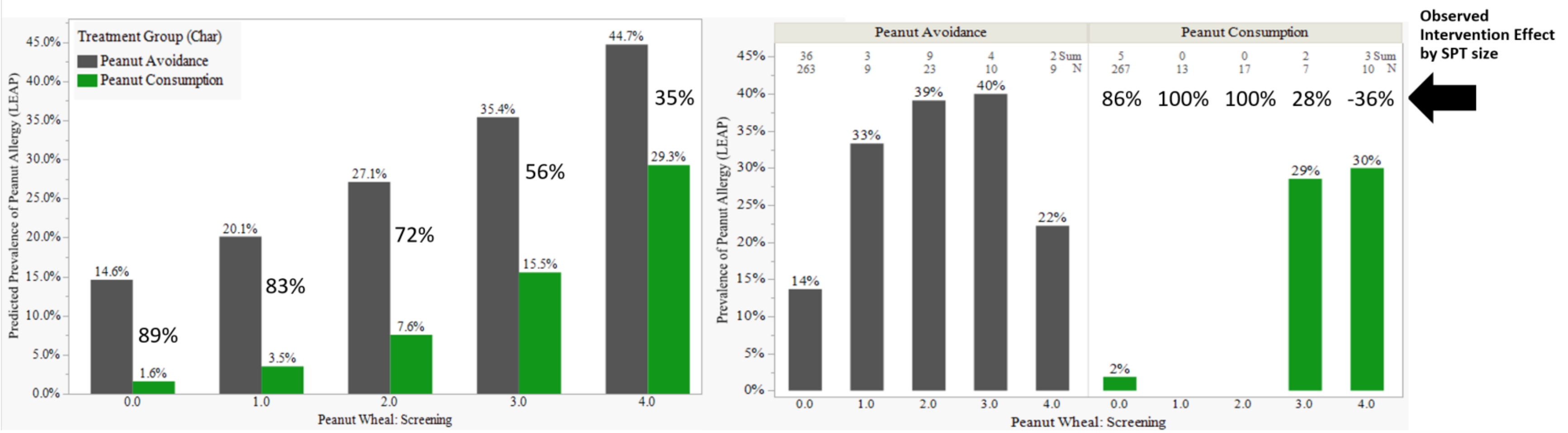

A logistic regression model was fit with baseline peanut SPT size (mm), randomized treatment assignment, and an interaction effect between the two. (A) The predicted probability of allergy at 60 months is estimated for each treatment group and each baseline SPT size in an intention to treat (ITT) analysis. The relative reduction in peanut allergy was calculated across the strata and displayed to the right of the peanut avoidance group bar. (B) Shows the raw data according to the intervention group and SPT size. The numbers annotated at the top of each panel are the number allergic in each stratum over the total sample size. The percent above each bar is the proportion with peanut allergy at the LEAP trial primary endpoint assessment at 60 months of age. Observed relative reductions of peanut allergy are displayed in the right panel of (B); note that the paradoxical negative relative reduction estimate (-36%) at 4mm is driven by the lower than expected allergy rate in the 4mm Avoidance Group (22%), and with only 9 and 10 subjects per arm, the 95% confidence interval around the -36% estimate is very wide and is statistically consistent with the 35% benefit seen in the model. Moreover, all three of the allergic subjects in the early introduction group at 60 months were already allergic at baseline and therefore never received the LEAP intervention. The model in (A) smooths the treatment benefit across SPT values; if instead, peanut introduction is assumed to have no benefit for SPT>2 mm, the relative reduction curves for the treatment effect by age of first introduction are similar to those shown in **Figure 3**. So, while with the existing data it is not possible to know whether the true relationship between SPT and benefit is closer to the smooth model shown in (A) or to a much sharper decline in benefit with SPT>2mm as suggested by the raw data counts, this distinction is essentially inconsequential to the decision of when to introduce peanut.

Modelling the proportion of the whole population with peanut allergic sensitization across firsts year of life

These propensity score weights were then applied in an ordinal logistic regression model to estimate the proportion of the whole population that would be expected to have peanut SPT wheal sizes of 0, 1, 2, 3, 4, or >4mm across each month of age in the first year of life under a peanut avoidance strategy (**Figure E3)**. Peanut allergy in EAT at 3 years of age was considered to be a surrogate for peanut allergy at age 5. Henceforth we will refer to peanut allergy at 5 years in our whole population model.

Estimation of the prevalence of allergy at 5 years as a function of baseline SPT and age when SPT was measured under peanut avoidance and early introduction

A second logistic regression model was used to estimate the prevalence of allergy at 5 years of life conditional on SPT size and age when SPT was measured in the first year of life, also under a peanut avoidance strategy (**Figure E3**). The LEAP intention to treat intervention effect was estimated using a third logistic regression model with an interaction effect between SPT size at enrolment (0-4mm) and randomized treatment assignment (**Figure E4**). A 0% intervention effect was used for the SPT >4mm group, assuming there is no benefit of early peanut introduction in this subgroup. These effects were applied to the probability of allergy at 5 years in the avoidance group for each SPT by age category; the overall risk reduction for each age was then calculated as a weighted average of the SPT distribution for that age.

Using observed point estimates from EAT, LEAP and PAS studies

A number of approaches were used to estimate the optimal timing to prevent peanut allergy to ensure the robustness of our relative reduction estimates, with varying degree of assumptions. The first four used observed point estimates from EAT and from the combined EAT, LEAP and PAS trial databases. These were used to ensure the robustness of the whole population analysis.

1. Observed intention to treat effect from the EAT trial, i.e. relative reduction in peanut allergy observed in the EAT ITT population.[5]
2. Observed per protocol effect from EAT trial as an example of the impact of the intervention with optimal adherence, i.e. the relative reduction in peanut allergy in the EAT per protocol population.[6]
3. Combined LEAP+PAS dataset; the relative reduction in peanut allergy was calculated using the LEAP ITT population combined with the PAS cohort; this LEAP+PAS dataset was not weighted to match EAT for this sensitivity analysis shown in **Figure 3A** (blue line and point estimates). As PAS was an observational study, a treatment effect was imputed. Two simple assumptions were made for this treatment effect imputation, namely, the single low risk allergic subject from Group I would have had their allergy prevented with intervention (there was only 1 observed allergic case) and secondly, no allergy would have been prevented in the high risk Group IV subjects (**Table E1**). In addition, the observed PAS control data and the imputed PAS treated data were each assigned a weight of 0.5, to maintain the appropriate LEAP to PAS ratio. While a completely raw data analysis could have been implemented using LEAP data alone, the high risk PAS Group IV cohort was deemed important to incorporate because it includes the participants who are likely to be most adversely impacted by delayed introduction. Risk reductions were calculated by baseline month of age, and a logistic regression model using a spline term for age was fit to provide smoothed estimates. As an additional sensitivity analysis, bootstrapped confidence intervals were applied to these data without imputing an intervention effect or applying any regression smoothing techniques among the blue points in **Figure 3A**.
4. EAT trial data using the LEAP intention to treat effect size. The LEAP ITT treatment effect was applied at 3 months and 12 months to the early and standard introduction groups respectively. The calculation of the relative risks used the observed distribution across SPT size categories at 3 and 12 months. Since baseline SPT was not measured in the avoidance group, the baseline SPT distribution from the early introduction group was used, which is valid given randomization should produce an approximately identical distribution prior to the intervention. The SPT distribution in the avoidance group was used for the 12 month assessment. Derivation of the probabilities of allergy conditional on SPT size assumes that the odds ratio comparing the odds of allergy at 3 and 12 months in EAT is the same as that in LEAP, and further use the fact that in the same these conditional probabilities are known in EAT for the avoidance group at 12 months. Applying the LEAP intervention effect to these rates gives an estimate of EAT allergy rates under the LEAP intervention, and these together can be used to estimate the relative reduction in allergy under the LEAP intervention. In

summary, with the assumption that the 3 month allergy rate is associated with the 12 month allergy rate by a constant odds ratio, this odds ratio could be determined to be consistent with the observed overall 3 year allergy rate. As EAT is a normal population, no population weights were applied.

Estimating the impact of early introduction of peanut to the whole population using EAT, LEAP and PAS study data

5. In this separate modelled approach, a whole population model, based on EAT, LEAP and PAS study data, was used to estimate the impact of early introduction of peanut.

<u>Estimation of the proportion of general population with each peanut SPT result</u>

From this weighted model, proportions in a general population with peanut SPT wheal sizes of 0, 1, 2, 3, 4 or >4mm during infancy was estimated (see ④in **Figure E1**). The peanut allergy rate at study end (3 and 5 years of life in EAT and LEAP respectively; we assume that the 3 year EAT data is a surrogate for the 5 year data and so focus on a 5 year peanut allergy outcome in this study) was estimated with a second logistic regression model that was conditional on age and SPT (see ⑤). Together, these two models gave estimates for the prevalence of peanut allergy under an avoidance strategy for the whole population and according to key risk strata (ethnicity and eczema severity).

<u>Estimation of the impact of early peanut introduction across the entire population</u>

Impact of early peanut introduction was assessed in the entire LEAP screening cohort of 834 participants. This took into account the 118 children who could not be randomized because their eczema was too mild (Group I) and the 76 children who could not be randomized into the trial because of a high likelihood of pre-existing peanut allergy (Group IV). To estimate the effect of the LEAP intervention across the whole screening population we made two key assumptions: (a) the effect of early introduction of peanuts in the diet is primarily dependent on the SPT size and (b) no treatment effect was assumed in Group IV (0% reduction in allergy) as the study design excluded participants with SPT sizes greater than 4mm since they were deemed already allergic (**Table E1** shows the complete set of assumptions and inherent limitations in combining the cohorts).[8] The LEAP intention to treat effect was estimated using a logistic regression model with an interaction effect between SPT size and randomized treatment assignment (see ⑥ in **Figure E1**). The raw data shown in **Figure E4B** show a sharper decline in efficacy for SPT of 3 mm or greater than the fitted logistic model. However, as a sensitivity analysis using the whole population evaluated under an alternate model of no benefit for these SPT values, the resulting curve for treatment benefit by age of introduction was essentially unchanged. Final predicted peanut allergy in the intervention group is shown in ⑦. All modeling used inverse probability weighting based on the propensity score in order to make the resulting weighted LEAP/PAS sample match the general population reflected in the EAT trial. The model of SPT outcome uses a proportional odds logistic regression approach incorporating the predictors' age, entered as a natural cubic spline with knots at 6 and 9 months of age, and randomized treatment group. The binary logistic regression model of allergy incorporates randomized treatment group, skin prick test outcome, and age (linear only effect) for overall predictions. Estimates for the different risk groups were made by including additional predictors (e.g. eczema severity and ethnicity) in the model of allergy.

Estimating the impact of early introduction of peanuts into the whole population

Estimates of relative reduction of allergy risk to determine the optimal timing of introduction was made using a two-stage modeling approach. This approach estimates the probabilities within a certain group of having membership in each SPT size category, conditional on age, and for the avoidance group, the probability of allergy given SPT size category and age. To find the analogous probability of allergy in the early introduction group the LEAP intervention effects were applied to the avoidance group. Overall allergy probabilities were then calculated by taking the dot product of appropriate SPT size category probabilities and allergy probabilities across SPT size categories. These overall estimates were then used to estimate the reduction in the risk of allergy associated with early peanut introduction relative to avoidance (see ⑧ and ⑨ in **Figure 1**).

Based on this two-stage approach, the estimated probability of allergy is calculated by summing across all SPT size categories of the product of the predicted proportion of the population at a certain SPT category and their respective allergy rate conditional on age. These probabilities are then used to estimate the reduction in the risk of allergy associated with early peanut introduction relative to avoidance; these curves are smooth because of the underlying models that produced them induced that smoothness.

Ninety-five percent confidence intervals for prediction using the two-stage approach are calculated using the bootstrap (with 10,000 replications). This is done by resampling the data stratified by study so that each bootstrap resample contains the same number of participants within a study as the original sample. Within each bootstrap resample all modeling steps were repeated, including the calculation of probability weights, estimation of the proportional odds

logistic regression model, estimation of the binary logistic model of allergy, and the estimation of allergy probabilities and relative risks. Pointwise 95% confidence bands for allergy as a function of age and allergy risk factors (namely SCORAD group and ethnicity) were then calculated as the 2.5 and 97.5 percentiles of the distribution of the bootstrap estimates.

In summary, the LEAP intervention effect by SPT shown in **Figure E4** was theoretically applied to the modeled SPT distribution at each month of age during infancy. The resulting relative reductions in peanut allergy were estimated from this two-stage model and displayed in **Figure 3**. The assumptions made are shown in **Table E1**.

H. Role of the funding source

The National Institute of Allergy and Infectious Diseases were involved in the study design and interpretation of this analysis. None of the other funders had any involvement with this analysis.

2. RESULTS

A. Participants from EAT and LEAP and PAS studies

The EAT, LEAP screening, LEAP and PAS study participants are described in **Figure E5**. Baseline details of the participants have been previously published,[2] as have the follow up details.[1,3,7]

The EAT trial participants had a mean age at enrolment of 14.7 weeks (range 12.9 to 18.0), 85% were of white ethnicity 3.2% black, 2.2% Asian, 0.8% Chinese and 9.2% mixed (mix of more than one ethnicity) ethnicity with an equal male-female split.[12] The LEAP trial participants were older at 4-11 months of age, 74% white, 7.5% black, 3.5% Asian, 1.1% Chinese and 13.6% mixed ethnicity with around two-thirds were male.[2] Where the participants differed at baseline was in terms of eczema: most (76%) EAT participants did not have eczema[6] while LEAP participants had an increasing proportion of severe eczema moving from LEAP group I to LEAP group IV.[2] Together these participants covered the entire range of eczema severity (**Figure E6**).

At enrolment into the studies in the first year of life, there were a total of 85 participants with peanut allergy or likely peanut allergy. These were mainly from the PAS study (n=76, cohort IV, excluded from the LEAP RCT) with others from LEAP RCT (n=7) and EAT (n=2, however, both were tolerant to peanut at 3 years of age).

In the EAT trial, a total of 15 of 597 (2.5%) participants in the standard introduction group had peanut allergy aged 36 months compared to 7 of 571 (1.2%) in the early introduction group (intention to treat group).[6] In LEAP group II, 36 of 263 (13.7%) participants randomised to the avoidance group compared to 5 of 266 (1.9%) participants in the early introduction group had peanut allergy at 60 months of age.[1] In LEAP group III, 18 of 51 (35.3%) participants randomised to the avoidance group and 4 of 41 (10.6%) participants in the early introduction group had peanut allergy at 60 months of age.[1] LEAP groups I and IV were not randomised to avoidance or early introduction; in group I a total of 1 of 47 (2.1%) participants had peanut allergy at 60 months compared to 48 of 59 (81.4%) participants in group IV.[1]

B. Factors associated with peanut allergy during the first year of life

Ethnicity, eczema severity and age or duration of eczema as key factors underlying the development of peanut allergy in the first year of life

A logistic regression analysis, taking into account both age and duration of eczema at screening, clearly shows that the latter is the most important determinant of early peanut allergy (**Figure E7**). However, given that age is a more accessible variable in a public health dimension than duration of eczema, we have chosen to use age as the key variable in this modelling.

The LEAP screening study population was 72.4% white, 13.1% mixed and 14.5% Asian/black/Chinese/other. The EAT study population was 84.7% white, 9.1% mixed and 6.1% Asian/black/Chinese/other. Non-Caucasian ethnicity predominately referred to Asian (mainly Indian subcontinent family origin) and Black (mainly Afro-Caribbean family origin). Combining the EAT and LEAP cohorts, non-white (including mixed) infants were estimated to have a higher likelihood of peanut allergy compared to white infants in the first year of life (relative risk=2.22, 95% confidence interval 1.45 to 3.33, p<0.001) (**Figure E9**).

Non-Caucasian ethnicity and presence, severity, and duration of eczema were all independently associated with an increased risk of peanut allergy in the first year of life (**Figures E8 and E9**). There is a window of opportunity to prevent peanut allergy in the first months of infancy irrespective of race or presence and severity of eczema.

Sensitivity analysis - time to event model

In addition to modeling with logistic regression, a population weighted, Weibull time to event model was developed to look at the risk of developing infant peanut allergy during the first year of life and its relation to eczema severity and ethnicity. This sensitivity analysis used SPT wheal size greater than 4 mm as a surrogate measure for allergy. All patients were assumed to be non-allergic at birth. The month at which a large wheal occurred or the participant left the study without experiencing a large wheal was recorded. The latter group of subjects was considered censored at the time they were lost to follow-up or were followed for 12 months. The analysis showed that there was significantly more rapid development of peanut allergy in infants with eczema (p<0.001). The estimates from this time to event model agreed with the logistic regression and the raw data shown in **Figure 2**. Specifically, the time to event model estimates that approximately 69% of the allergy burden developed by 12 months of age with significantly more peanut allergy developing in those with non-white ethnicity and eczema (**Figure E9**).

Figure E5. Consort figure showing LEAP screening, LEAP and EAT studies

(A) LEAP screening and randomized controlled studies (SPT-negative and SPT-positive groups)

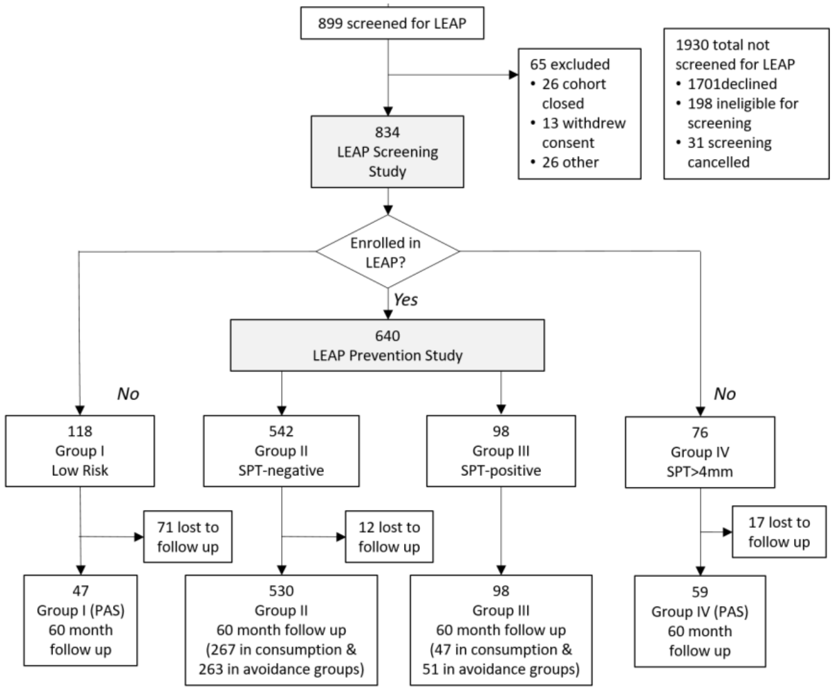

(B) EAT randomized controlled trial

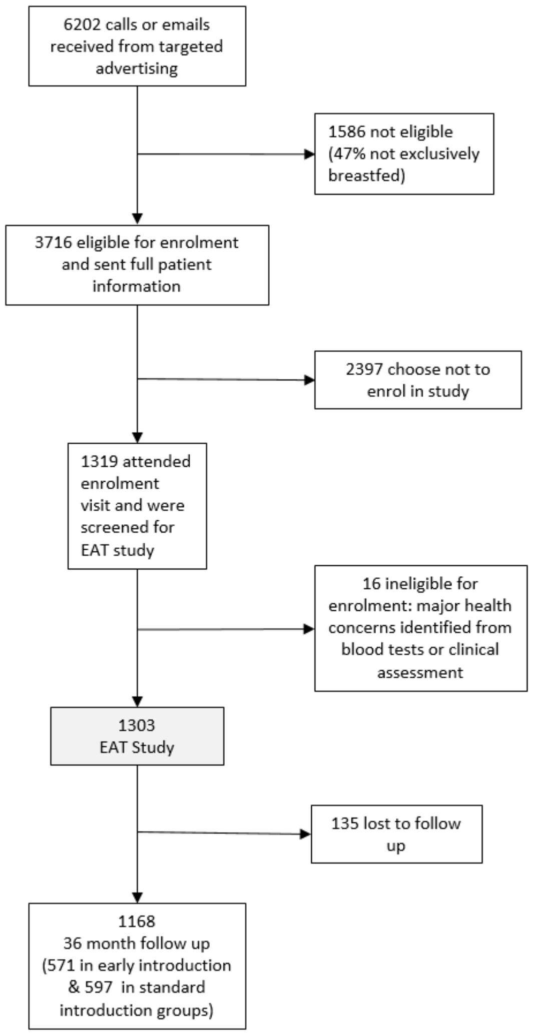

These consort figures describe the participant flow through the LEAP (A) and EAT (B) trials. The LEAP screening study also had a group V (n=65) with a miscellaneous group of participants (e.g. cohort already full) who were not followed up to 60 months and so are not included in this analysis.

Figure E6. Distribution of infants with no, mild, moderate and severe eczema in each study population

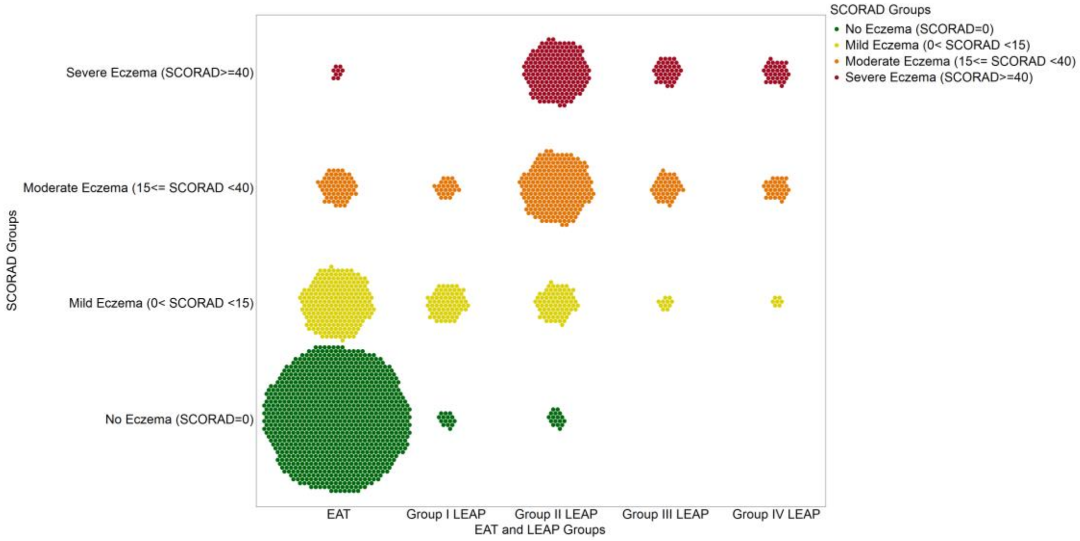

Participants included in EAT trial and LEAP screening study Groups I-IV. Each dot represents a single participant. Participants in each study are divided into no (green), mild (yellow), moderate (orange) or severe (red) eczema based on their SCORAD assessment. Between the EAT and LEAP studies, there is good coverage of the different severities of eczema in the general population. SCORAD was assessed at the baseline assessment for participants in EAT (3 months) and LEAP (4-11 months) study Groups I, II, III and IV.

Figure E7. Combined effect of eczema severity and duration and age on the likelihood of peanut allergy at the screening visit in LEAP

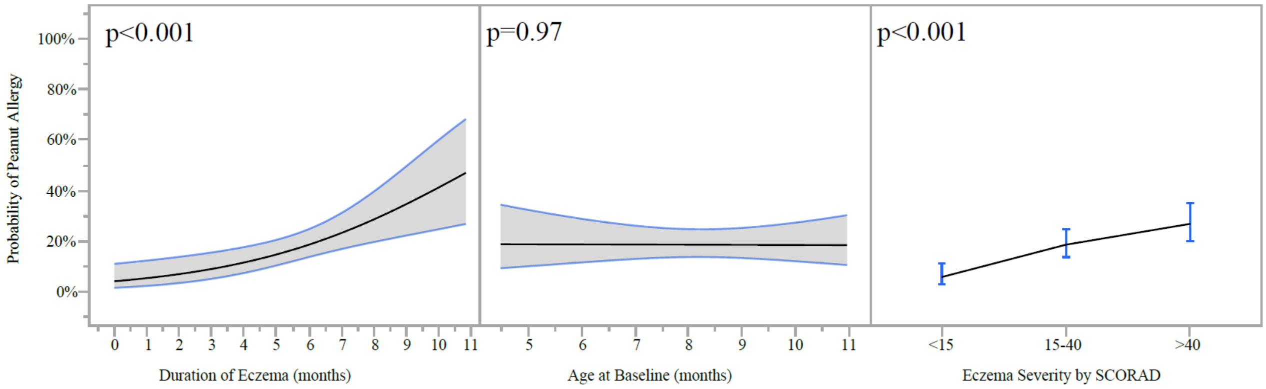

The regression lines show probability curves for peanut allergy at the LEAP screening visit. The curves are mutually adjusted for each variable in the multivariable logistic regression model (age at screening, duration of eczema at screening, and eczema severity based on SCORAD. Data are from the LEAP screening study groups I and IV, and the early introduction arm only of groups II and III, as the subjects randomized to consume were first given a baseline oral food challenge. Group I participants were assumed to be tolerant at baseline and group IV assumed to be allergic (peanut SPT >4mm). The odds ratio (95% confidence interval) of peanut allergy versus no allergy for a one month increase in the duration of eczema was: 1.3 (1.1, 1.6).

Figure E8. Relationship between baseline duration of eczema and age with baseline peanut allergy status during the first year of life

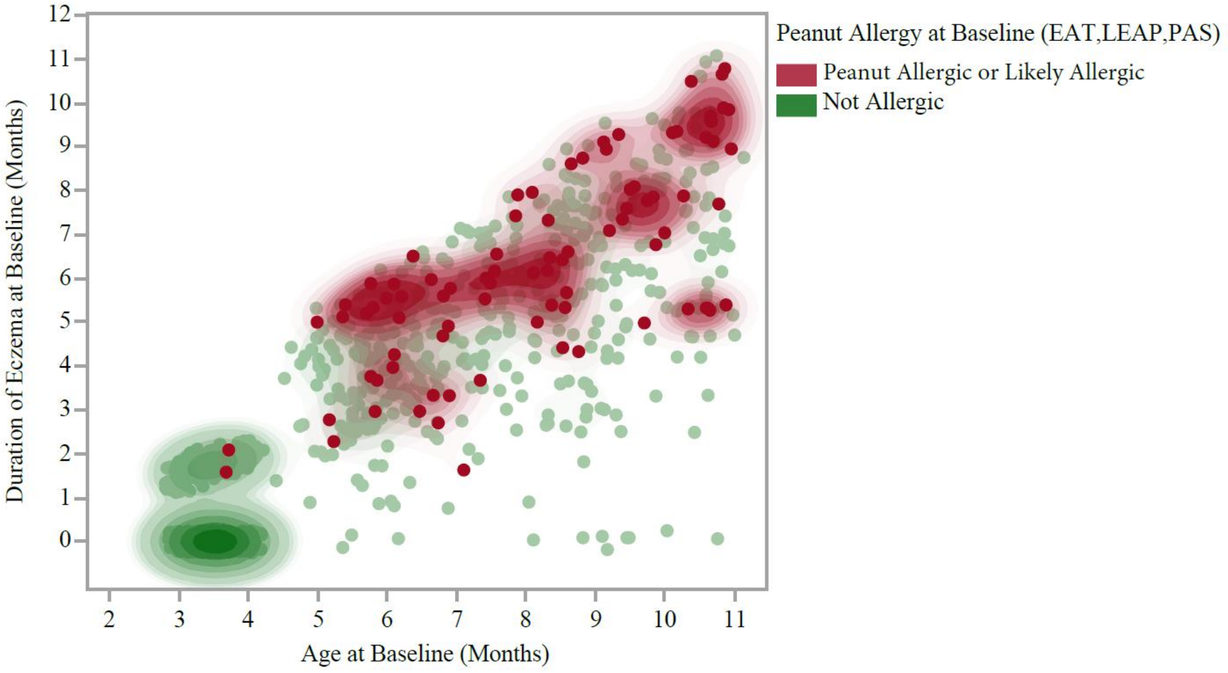

Age, duration of eczema, and peanut allergy presented as a contour plot, with all points jittered to prevent over-plotting. EAT data shown on lower left quadrant for comparison to LEAP and PAS. EAT ages are randomly imputed between 3 and 4 months (age at enrolment) and participants with eczema had durations imputed based on half the age at baseline plus random uniform jitter (actual durations of eczema not available). Age and duration of eczema in LEAP and PAS are actual values, not imputed. Peanut allergic or likely allergic (red) participants defined as: LEAP screening Group I - all assumed to be tolerant; Groups II and III – early introduction only – defined according to baseline peanut challenge (n=7 allergic); and Group IV – all assumed to be likely peanut allergic (n=76) as peanut wheal >4mm at baseline; EAT – early introduction only – defined according to baseline peanut challenge result (n=2 allergic). Contour plots were created with nonpolar densities to visualize the concentration of allergy present in older ages and longer durations of eczema within the LEAP and PAS participants. Both participants defined as allergic to peanut at 3-4 months were from the EAT study and were tolerant to peanut at 3 years of age.[6]

Figure E9. Time to event modeling of the development of allergy in the first year of life based on SPT >4mm

(A) **(B)** **(C)**

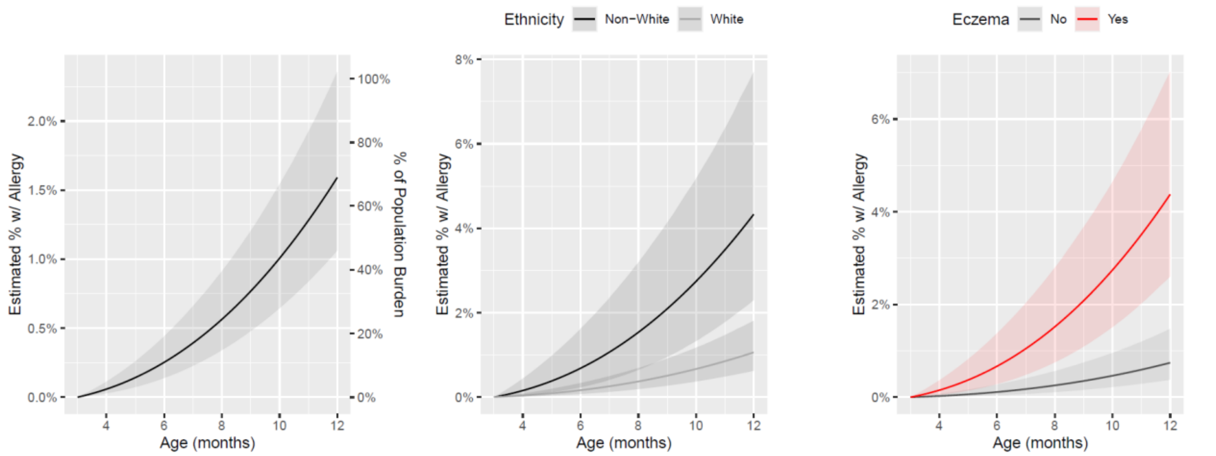

Figures show time to event modeling of the development of peanut allergy (defined as wheal >4mm) in (A) all, (B) by white and non-white ethnicity (including mixed) and (C) by eczema. Lines represent estimated percentage with peanut allergy at each age while shaded areas represent 95% confidence interval. The right y-axis in panel A is shown for reference and is computed based off the previously modeled estimated allergy prevalence of 2.3% at 60 months (e.g. at 12 months the allergy rate is estimated to be 1.6%, which when divided by 2.3% gives shows that approximately 69% of the population allergy burden occurs in the first year of life). All participants in the EAT, LEAP, and PAS studies were included (n= 2137). Analysis used a propensity score weighted Weibull time to event model, and incorporates interval censoring where needed. A SPT wheal size greater than 4 mm was used as a surrogate measure for allergy, since allergy status was only recorded at the end of the study (3 and 5 years of age). All participants were assumed non-allergic at birth. The month at which a large wheal occurred or the participant left the study without experiencing a large wheal was recorded. The latter group of subjects was considered censored at the time they were lost to follow-up or were followed for 12 months.

Figure E10. Distribution of peanut wheal diameters by age in the first year of life

(A)

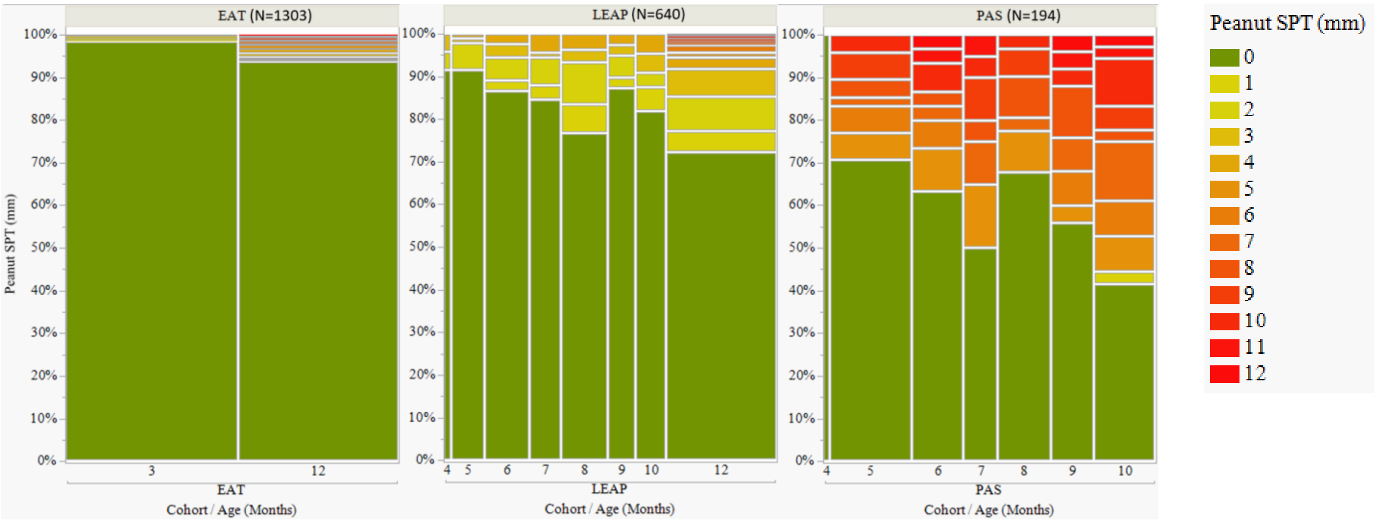

(B)

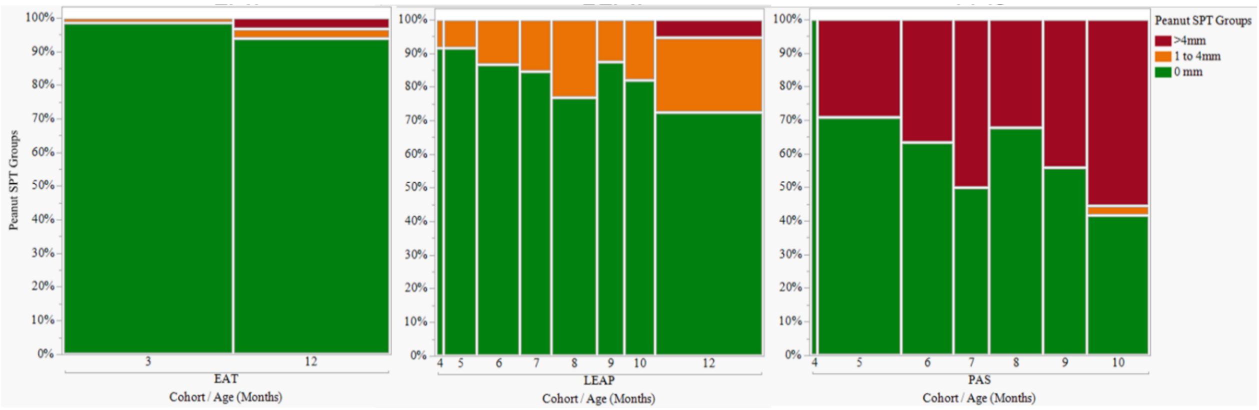

The stacked bars represent the proportion of participants with peanut wheal diameters (A) 0mm to 12mm (raw data) and (B) 0, 1-4 and >4mm by age in months at the baseline visit and 12 month visit. The bar width in each panel represents the proportion within that group. The first panel presents participants from the EAT intervention group at 3 months (baseline) and the control group at 12 months (SPT was not collected in the control group at 3 months in EAT). The second panel presents all participants from the LEAP study (LEAP screening Groups II-III) at their baseline visit by age at screening; the 12 month data only displays LEAP avoidance group participants since the intervention significantly reduced SPT size. Therefore, in order to visualize the distribution of wheal sizes in infancy among participants not consuming peanut, repeated measurements are shown at baseline and 12 months for the LEAP Avoidance Group. The third panel presents all participants from the PAS study (LEAP screening Groups I and IV) according to their age at screening (a 12 month assessment was not done in the PAS cohort).

C. **Estimating the impact of early introduction of peanuts to the whole population**

Potential impact of applying the LEAP intervention to EAT, a normal risk population

Intervention effectiveness is similar across all eczema severities

Analysis of the LEAP trial data demonstrated that the percentage reduction in peanut allergy with early introduction varied with peanut wheal size (**Figure E4**) but was similar with different eczema severities (**Figure E11**).

Estimating the impact of early introduction at different ages to the whole population

In order to estimate the impact across the whole population, a predictive model was generated for peanut allergy at 60 months using the LEAP, PAS and EAT intention to treat populations (**Figures E3A and E3B**).

Intervention effectiveness decreases with increasing SPT diameter

The following relative reductions in peanut allergy were derived for each SPT group from 0, 1, 2, 3, 4, >4mm respectively: 89%, 83%, 72%, 56%, 35%, 0% (intention to treat population) as described in the methods and **Figure E4A**). To overcome the issue of small numbers in the 3mm and 4mm categories, a logistic regression model with an interaction effect between SPT size and randomized treatment assignment was generated using the intention to treat population. This provided estimates of the intervention size in each SPT category (**Figure E4A**).

Estimating optimal timing to prevent peanut allergy using observed point estimates from EAT, LEAP and PAS studies

A number of approaches were used to estimate the optimal timing to prevent peanut allergy to ensure the robustness of our relative reduction estimates. The first four used observed point estimates from EAT and from the combined LEAP and PAS trial databases and do not require the weights of the combined cohorts. However, as described earlier, the third incorporated imputation for a treated PAS effect, and the fourth applied the LEAP treatment effect to EAT data, and required additional assumptions to estimate the probability of allergy given SPT at 3 months under avoidance. Tabular data underpinning values shown in the figures are shown in **Tables E4-6**.

1. Observed intention to treat effect from intervention effects from EAT trial was around 50% (red square, **Figure 3A**). As EAT participants were randomized at 3 months of age, this gives the intention to treat effect size around that age. No assumptions required.
2. Observed per protocol effect from EAT trial as an example of the impact of the intervention at 3 months of age with optimal adherence (top red square, **Figure 3A**). Within a per protocol situation, the relative reduction was 100%. No assumptions required.
3. Combined LEAP+PAS dataset (blue circles and blue line, **Figure 3A**) with imputed treatment effect in PAS cohort; chiefly that there is no treatment benefit for SPT > 4 mm. The smoothed regression line shows how the relative reduction in peanut allergy decreases with increasing age of introduction of peanuts into the infant diet in a high risk population. An additional sensitivity analysis with bootstrapped confidence intervals (**Figure E13**) demonstrates the significant reduction in effectiveness of the intervention as its introduction is increasingly delayed over the first year of life.
4. EAT trial data using the LEAP effect size (red circles and red dotted line, **Figure 3B**). By applying the LEAP ITT effect size to modeled 3 month data from early introduction group and to raw 12 month data from standard introduction group, the relative reduction in peanut allergy can be seen to drop from around 80% to around 20%. A considerable reduction with delaying of the introduction of peanut into the infants' diet.

All these analyses, using observed point estimates, give similar conclusions with the relative reduction in peanut allergy being highest when applied earlier in life. Additionally, they give similar conclusions to the whole population model (**Figures 3B, 3C and Figure E12**).

6. Whole population model composed of normalized LEAP+PAS data set plus EAT (black line with bootstrapped confidence interval, Figure 3B). This approach relies on more assumptions such as the weighting approach needed to integrate the data sets to represent a whole population and assumed that the distribution of SPT in the avoidance group was similar to the intervention one (**Table E1**).

The result from our whole population model are consistent with our time to event modelling (**Figure E7**), which shows a significantly more rapid development of peanut allergy over the first 12 months of life in infants with eczema ($p<0.001$) and of non-white ethnicity ($p<0.001$).

Figure E11. Relative reduction in peanut allergy with LEAP intervention by SCORAD group

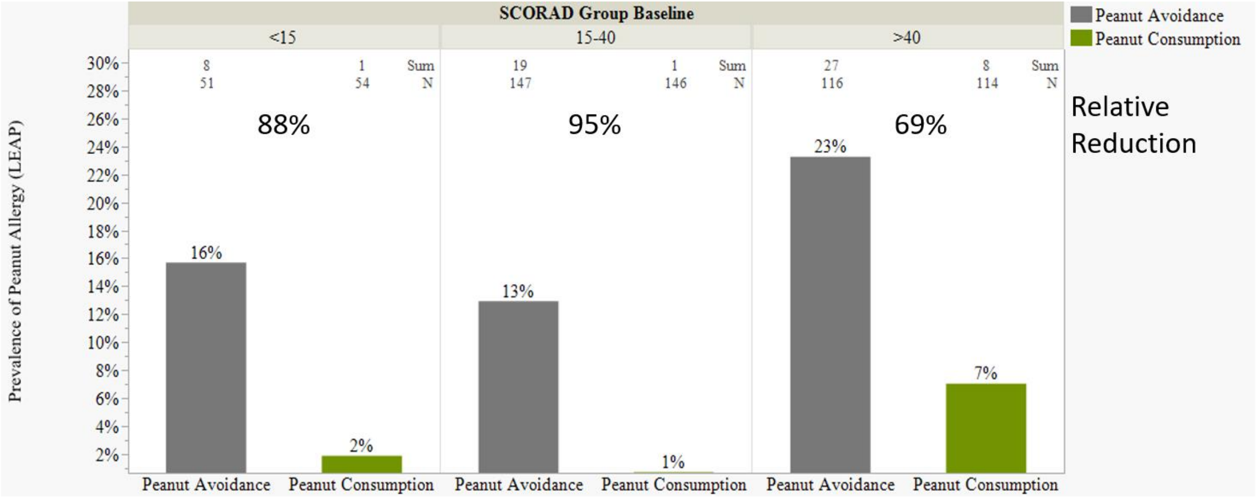

The observed ITT subgroup effect sizes are displayed for each SCORAD group (eczema severity) at baseline. Unlike with peanut SPT size, no clear trend was evident with respect to the treatment effect by SCORAD groups.

Figure E12. Population modeled ITT relative reductions in peanut allergy and bootstrapped 95% confidence intervals for (A) all participants and (B) by eczema severity, and (C, D) by ethnicity

(A)

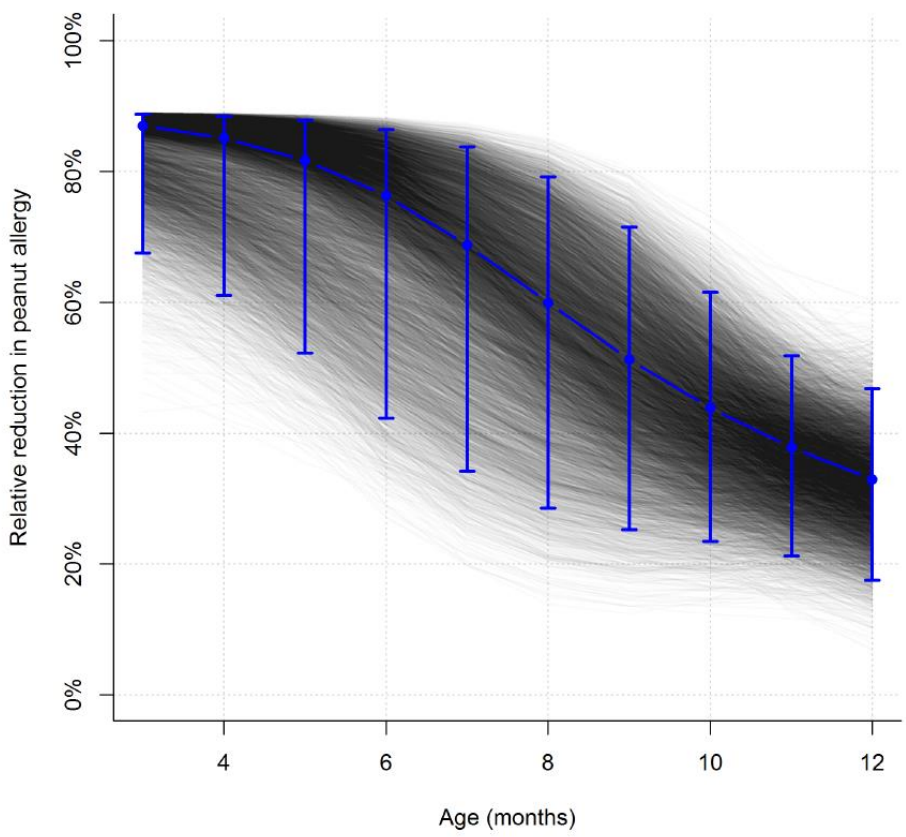

(B)

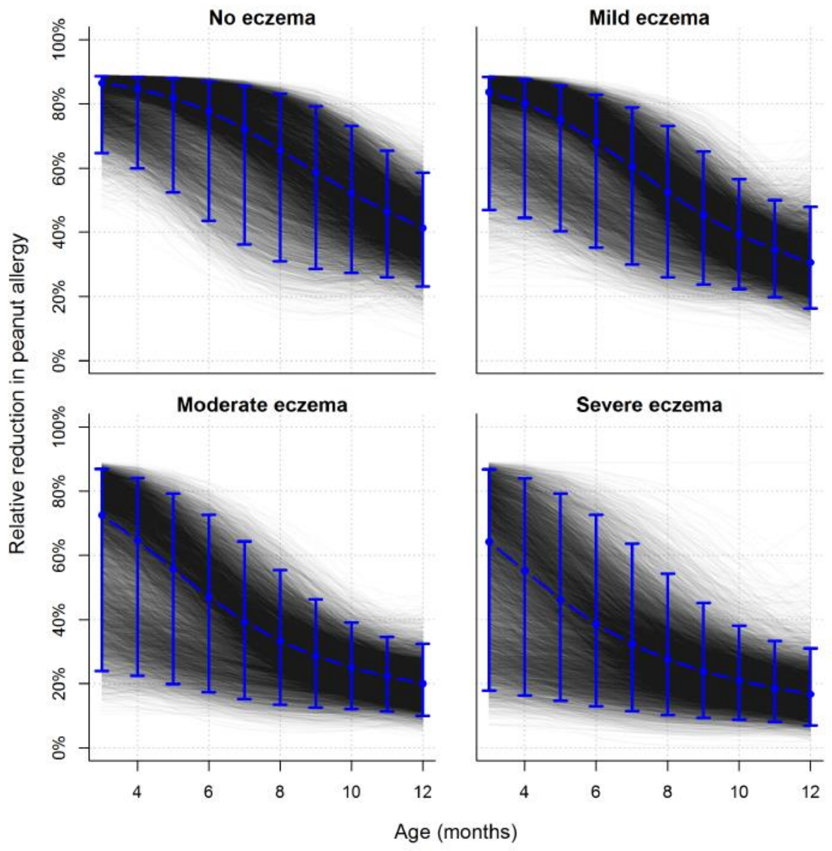

(C)

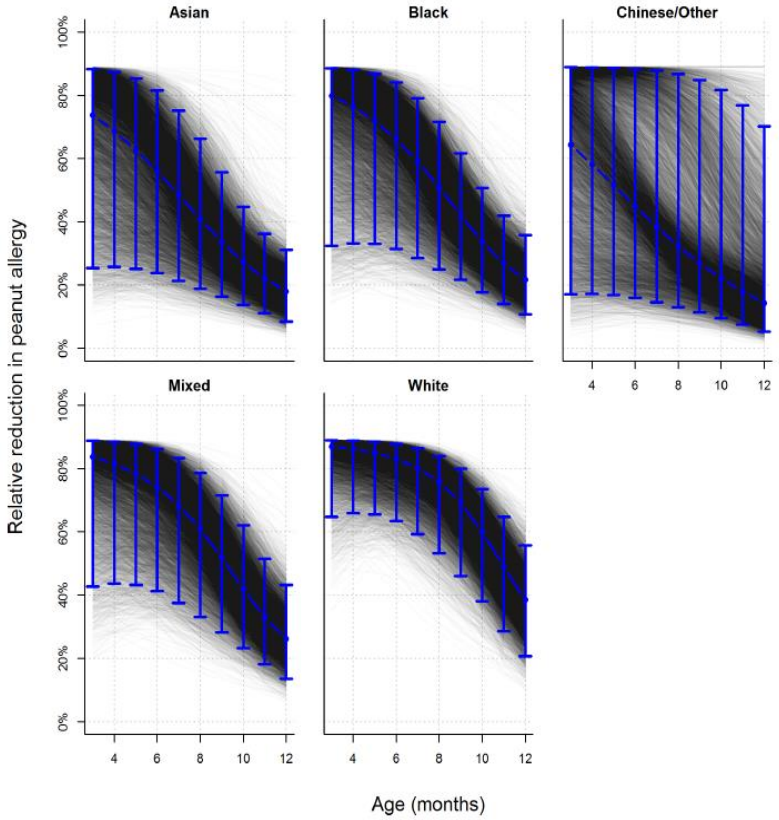

(D)

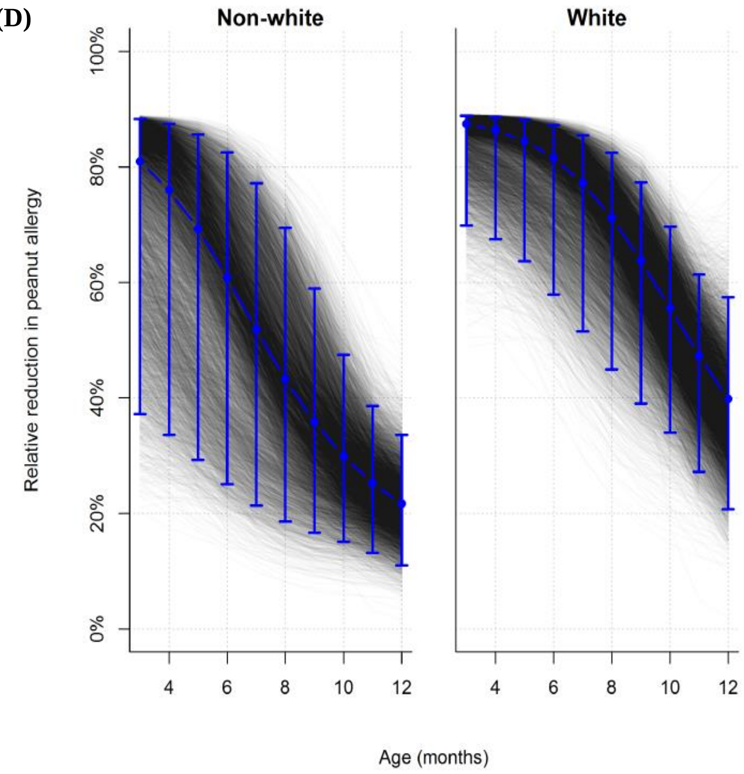

These estimates of the relative reduction in risk of peanut allergy with the early introduction of peanuts in the diet at different ages are produced by a two-stage modeling approach using bootstrapping to enable the precision of the model estimates to be calculated (summarized on **Figure E1**). Using EAT as the reference population, a propensity score weighted logistic regression model using eczema severity, ethnicity and egg allergy was used to estimate the proportions in a normal population expected to have peanut SPT wheal sizes of 0, 1, 2, 3, 4 or >4mm during each month of infancy. From these estimated proportions, the peanut allergy rate at 5 years of life was estimated with a second logistic regression model. Together, these two models give estimates for the prevalence of peanut allergy under an avoidance strategy. Finally, The LEAP intervention effect by SPT shown in **Figures E7 and E11** was theoretically applied to the SPT distribution at each month of age during infancy. The resulting relative reductions in peanut allergy are estimated from this two-stage model and are shown for the overall normal population (A) and according to key risk strata of eczema severity (B) and ethnicity (C and D). The point estimates from this modelling approach are shown as a blue line and estimates from each individual bootstrap sample are in light grey. The pointwise 95% confidence intervals are based on the bootstrap estimates (see Section G, Estimating the impact of early introduction of peanuts in the whole population, for details).

Figure E13. Estimated relative reduction in peanut allergy and bootstrapped 95% confidence intervals in the LEAP and PAS cohorts

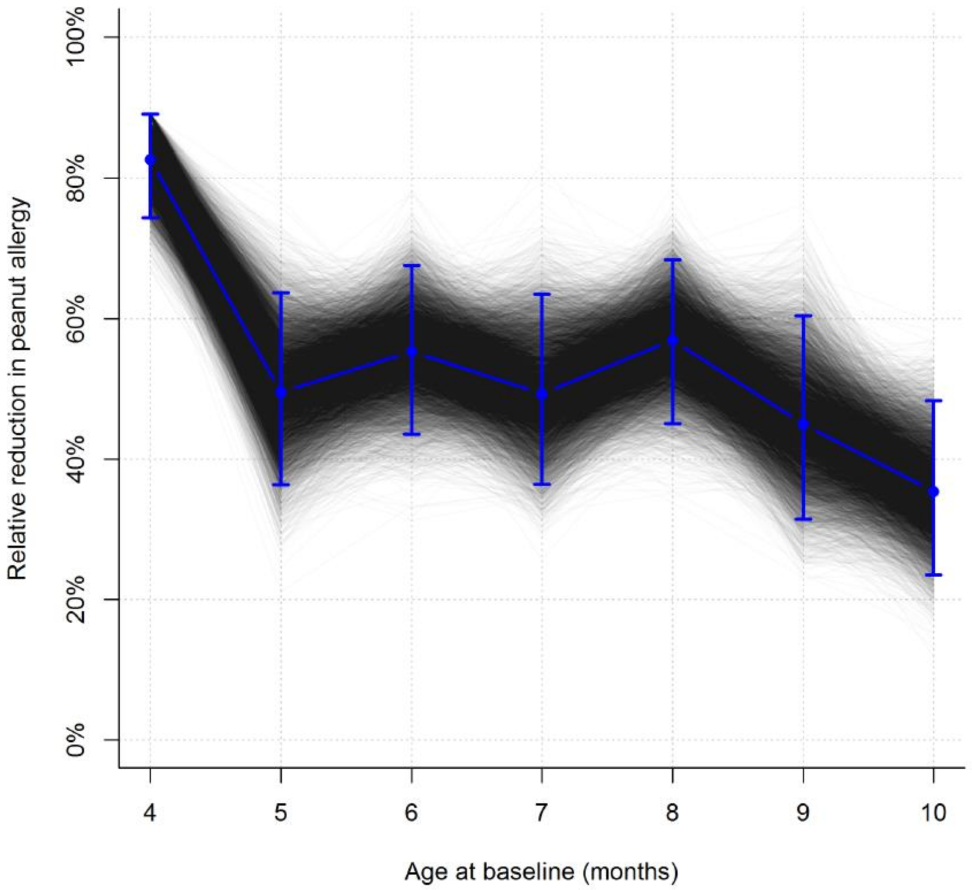

This sensitivity analysis was performed to provide raw relative reduction estimates in a high risk population without applying the modeling assumptions used in **Figure 3** and **Figures E12 (A-D)**. Specifically, the bootstrapped estimates are calculated directly from the observed allergy outcomes at 60 months of age in the combined LEAP trial and PAS dataset. The model uses the distribution of SPT at each age and applies the LEAP intervention effect (**Figure E4**) as a function of SPT at each age. The point estimates are shown as a blue line and estimates from each individual bootstrap sample are in light grey. The pointwise 95% confidence intervals are based on bootstrapped estimates without applying propensity score weighting (see **supplementary Methods Section G**, Estimating the impact of early introduction at different ages to the whole population - combined LEAP+PAS dataset, for details). This means that the population distribution is not representative of the general population but instead of this high risk population. Note that the bootstrapped estimates of variability sample from each age which have small sample size for some of the age groups (e.g. there are only 28 subjects at 4 months of age). The bootstrap can give poor estimates of variability when sample sizes are small; nevertheless, this analysis uses the observed distribution of the data without making the modeling assumptions used in **Figure 3**.

Figure E14. Sensitivity analyses of different approaches used to weight LEAP+PAS to a normal population (EAT)

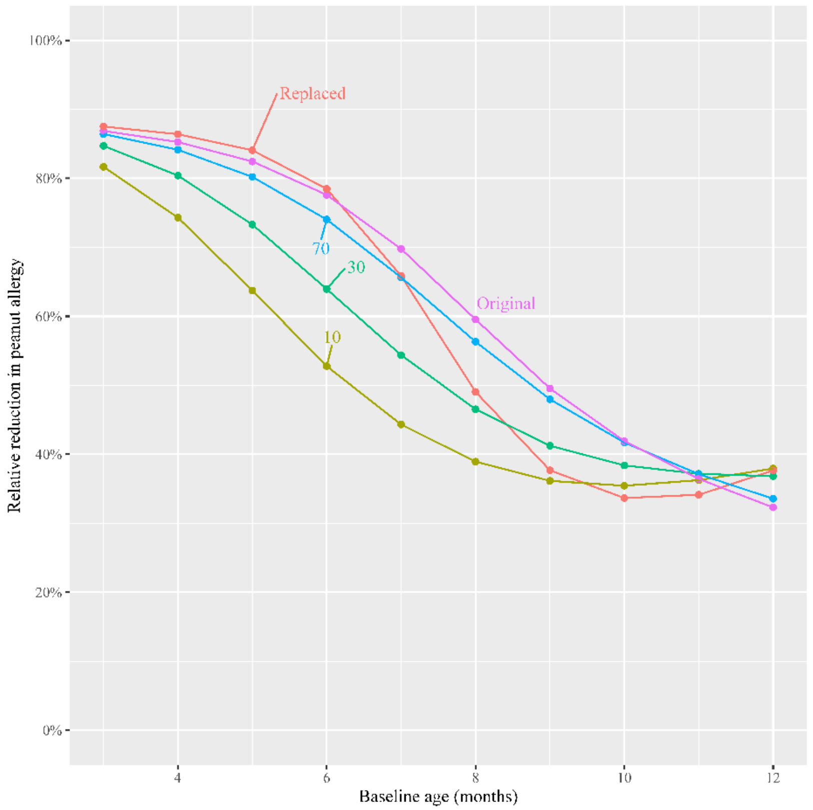

Weights for the LEAP/PAS cohort were computed using propensity scores with a goal of matching the distribution seen in the EAT cohort on baseline egg allergy, eczema severity and ethnicity (see **Figure E2** for details). Some individuals in the LEAP/PAS cohort were assigned very large weights, and thus this sensitivity analysis was done to consider how the results would have changed under different weighting approaches. The estimated relative reduction in peanut allergy is displayed using different weighting strategies to trim or replace subjects with large weights in the LEAP and PAS cohorts. The "Original" line represents the estimates under the primary weighting strategy used in **Figure 3**. The other curves represent relative reductions under different methods of down-weighting highly weighted cases and redistributing their total excess weight to the other observations. The "Replaced" line represents a strategy where highly weighted observations from LEAP/PAS are completely removed from the dataset and their total weight distributed across similar subjects in EAT (i.e. those with no egg allergy, White ethnicity, no eczema). The relative reduction curves labeled with numbers represent commonly used trimming strategies, where cases with weights above a given cut-off value (given by each curve label), are trimmed to have new weights equal to that cut-off value. Trimmed weights were calculated using functionality within the R package *survey*.[14] Under this strategy, the total amount of trimmed weight is reassigned to the other observations in an amount proportional to their original weights. This process is applied recursively until all weights are at or below the cut-off. As can be seen by comparing the lines representing sensitivity analyses, trimming or replacing LEAP and PAS subjects with other subjects in the dataset creates a higher risk cohort and results in relative reduction curves that are generally lower. This sensitivity analysis again illustrates there is some uncertainty about the shape of the risk reduction curve between 3 and 12 months, but the advantage of early introduction is seen regardless of which approach is used.

Figure E15. Association between infant peanut allergy and eczema severity and egg allergy in the first year of life (LEAP/PAS) and 1 year of age (EAT)

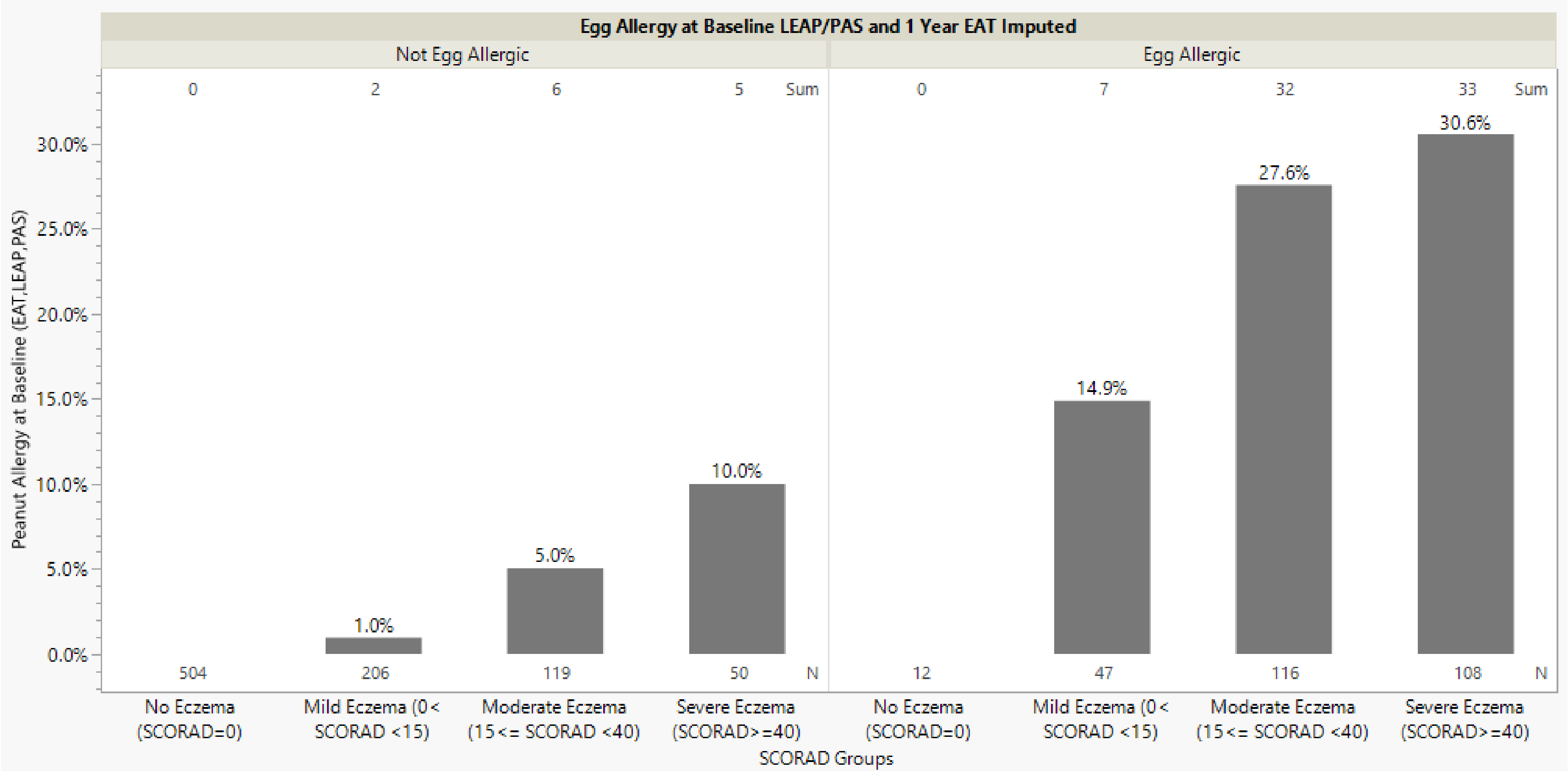

The prevalence of peanut allergy at baseline is shown by eczema severity and stratified by the presence of egg allergy at baseline in LEAP/PAS and at the year 1 assessment in EAT, illustrating an independent association of both eczema severity and egg allergy with peanut allergy at baseline. The number with peanut allergy is annotated at the top of the bars and the sample size in each group is annotated below the bars. Subjects with missing egg allergy assessments in EAT were imputed as non-allergic.

Table E3. Estimates of relative reductions in peanut allergy based on scenarios with different timings of intervention

		Age of introduction							
		**All: 4 months**		**Eczema: 4 months No eczema: 6 months**		**Eczema: 4 months No eczema: 12 months**		**All: 12 months**	
**Eczema status**	**Proportion of the population**	**RR reduction estimate**	**When peanut introduced**	**RR reduction estimate**	**When peanut introduced**	**RR reduction estimate**	**When peanut introduced**	**RR reduction estimate**	**When peanut introduced**
None	76%	84.7%	4 months for all infants	77.6%	6 months	40.4%	12 months	40.5%	12 months for all infants
Mild	18%	80.2%		80.2%	4 months if any eczema	80.2%	4 months if any eczema	29.9%	
Moderate	4.9%	64.7%		64.7%		64.7%		19.6%	
Severe	0.5%	55.3%		55.3%		55.3%		16.4%	
**All infants**	**100%**	**82%**		**77%**		**58%**		**33%**	

Numbers represent proportion of the population and the relative risk (RR) reduction; results for all infants represents a weighted average, of the difficult subpopulations. Values for estimated reduction in peanut allergy are calculated from the model that underpins **Figure 3C** (that is, the relevant probabilities of allergy under an avoidance strategy used in these calculations were derived from that model). Eczema status defined according to SCORAD during infancy: none, 0; mild, 1-14; moderate, 15-40; severe, >40. Scenario 1, peanuts introduced into the diet of all infants at 4 months; Scenario 2, peanuts introduced into the diet of infants with eczema at 4 months and infants without eczema at 6 months; Scenario 3, peanuts introduced into the diet of infants with eczema at 4 months and infants without eczema at 12 months. **Table E3** uses the model from **Figure 3C**, this is different from the approach used in **Table 2** so results are slightly different although comparable.

Table E4. EAT observed allergy rates (ITT and PP)

	Early introduction	Standard introduction	Reduction
ITT	1.2 (7/571)	2.5 (15/597)	52%
PP	0 (0/310)	2.5 (13/523)	100%

Numbers represent percentages with peanut allergy (numbers with peanut allergy / total number in each group) at 36 months.
These numbers relate to the EAT data points in **Figure 3A**.

Table E5. LEAP + PAS raw and imputed allergy rates (ITT) and estimated risk reduction by age of randomization

Age at Baseline	PAS Group I*		LEAP		PAS Group IV*		Overall		Risk Red'n
	Avoid**	Consume***	Avoid**	Consume**	Avoid**	Consume****	Avoid	Consume	
4	0 (4)	0 (4)	41.7 (12)	0 (11)			35.7 (14)	0 (13)	100%
5	2.9 (34)	0 (34)	22 (41)	5.9 (51)	71.4 (14)	71.4 (14)	22.3 (65)	10.7 (75)	52%
6	0 (19)	0 (19)	11.9 (67)	1.7 (58)	90.9 (11)	90.9 (11)	15.9 (82)	8.2 (73)	48%
7	0 (10)	0 (10)	16.7 (42)	0 (46)	70 (10)	70 (10)	20.2 (52)	6.2 (56)	69%
8	0 (21)	0 (21)	18.2 (66)	6 (67)	100 (10)	100 (10)	20.9 (81.5)	10.9 (82.5)	48%
9	0 (14)	0 (14)	7.5 (40)	0 (38)	72.7 (11)	72.7 (11)	13.3 (52.5)	7.9 (50.5)	41%
10	0 (16)	0 (16)	21.7 (46)	4.7 (43)	85 (20)	85 (20)	28.9 (64)	17.2 (61)	40%

Each entry is proportion allergic at 60 months (total number of participants). Figures relate to the LEAP + PAS curve in **Figure 3A**.
* Weighted 0.5; ** Raw data; *** Imputed to assumed total benefit; **** Imputed to assumed zero benefit of intervention.

Table E6. EAT modeled allergy rates (ITT)

SPT	3 months age			12 months age		
	Distribution in avoidance group++	Modeled Allergy Rate		Dist'n Avoid	Modeled Allergy Rate	
		Avoid**	Consume+		Avoid*	Consume+
0	98.6	1.9	0.2	93.8	0.4	0
1	0.2	8.5	1.4	0.3	1.6	0.3
2	0.6	27.4	7.7	0.5	6.4	1.8
3	0.3	53.7	23.6	1.2	17.3	7.6
4	0.3	72.8	47.3	0.8	32.6	21.2
>4	0.0	84.3	84.3	3.3	49.2	49.2
Overall		2.5	0.5		2.5	1.9
Relative Risk Reduction	81%			21%		

Figures are frequency distribution of SPT results in avoidance group and then proportions with peanut allergy at 3 and 12 months.
* 36 month allergy rate in avoidance arm conditional on 12 month SPT is based on observed data in EAT's delayed introduction arm, but smoothed with logistic; ** 36 month allergy rate in avoidance arm conditional on 3 month SPT is not observed. Therefore, it is based on a model assuming constant odds ratio between 3 and 12-month allergy rate conditional on SPT, conditional on the overall allergy rate in avoiders being 2.5%; + EAT early introduction arm estimates are computed from LEAP treatment effect applied at 3 months; ++ Avoidance arm SPT distribution is imputed from the early introduction arm SPT distribution (which should be equal at baseline as a result of randomization). These data relate to the EAT curve in **Figure 3B**.

5. References

1. Du Toit G, Roberts G, Sayre PH, Bahnson HT, Radulovic S, Santos AF, et al. Randomized trial of peanut consumption in infants at risk for peanut allergy. New England Journal of Medicine. 2015;372(9):803-13.
2. Du Toit G, Roberts G, Sayre PH, Plaut M, Bahnson HT, Mitchell H, et al. Identifying infants at high risk of peanut allergy: the Learning Early About Peanut Allergy (LEAP) screening study. Journal of Allergy & Clinical Immunology. 2013;131(1):135-43.e1-12.
3. Du Toit G, Sayre PH, Roberts G, Sever ML, Lawson K, Bahnson HT, et al. Effect of avoidance on peanut allergy after early peanut consumption. New England Journal of Medicine. 2016;374(15):1435-43.
4. Kunz B, Oranje A, Labreze L, Stalder J-F, Ring J, Taieb A. Clinical validation and guidelines for the SCORAD index: consensus report of the European Task Force on Atopic Dermatitis. Dermatology. 1997;195(1):10-9.
5. Kelleher M, Dunn-Galvin A, Hourihane JOB, Murray D, Campbell LE, McLean WI, et al. Skin barrier dysfunction measured by transepidermal water loss at 2 days and 2 months predates and predicts atopic dermatitis at 1 year. Journal of Allergy and Clinical Immunology. 2015;135(4):930-5. e1.
6. Perkin MR, Logan K, Tseng A, Raji B, Ayis S, Peacock J, et al. Randomized Trial of Introduction of Allergenic Foods in Breast-Fed Infants. New England Journal of Medicine. 2016;374(18):1733-43.
7. de Silva D, Geromi M, Halken S, Host A, Panesar SS, Muraro A, et al. Primary prevention of food allergy in children and adults: systematic review. Allergy. 2014;69(5):581-9.
8. Peters RL, Allen KJ, Dharmage SC, Tang ML, Koplin JJ, Ponsonby AL, et al. Skin prick test responses and allergen-specific IgE levels as predictors of peanut, egg, and sesame allergy in infants. Journal of Allergy & Clinical Immunology. 2013;132(4):874-80.
9. Koplin JJ, Peters RL, Dharmage SC, Gurrin L, Tang ML, Ponsonby AL, Matheson M, Togias A, Lack G, Allen KJ, Allen K. Understanding the feasibility and implications of implementing early peanut introduction for prevention of peanut allergy. Journal of Allergy and Clinical Immunology. 2016;138:1131-41.
10. Sporik R, Hill D, Hosking C. Specificity of allergen skin testing in predicting positive open food challenges to milk, egg and peanut in children. Clinical & experimental allergy. 2000;30(11):1541-6.
11. Roberts G, Lack G. Food allergy--getting more out of your skin prick tests. Clinical & Experimental Allergy. 2000;30(11):1495-8.
12. Perkin MR, Logan K, Marrs T, Radulovic S, Craven J, Flohr C, et al. Enquiring About Tolerance (EAT) study: feasibility of an early allergenic food introduction regimen. Journal of Allergy and Clinical Immunology. 2016;137(5):1477-86. e8.
13. Rosenbaum PR, Rubin DB. The central role of the propensity score in observational studies for causal effects. Biometrika. 1983;70(1):41-55. doi:10.1093/biomet/70.1.41
14. T. Lumley. Analysis of complex survey samples. Journal of Statistical Software 2004;9(1): 1-19.
